# Role of Chronic Conditions in Out-of-Pocket Costs for Preventive Care in the US

**DOI:** 10.1001/jamanetworkopen.2025.53157

**Published:** 2026-01-08

**Authors:** Allan Tran, Audrey Laporte, Eric Nauenberg, Alex Hoagland

**Affiliations:** 1 Institute of Health Policy, Management and Evaluation, Dalla Lana School of Public Health, University of Toronto, Toronto, Ontario, Canada; 2 Canadian Centre for Health Economics, University of Toronto, Toronto, Ontario, Canada

## Abstract

**Importance:**

The Patient Protection and Affordable Care Act (ACA) requires private health insurers to cover recommended preventive services with no patient cost-sharing, but patients covered by these provisions still incur out-of-pocket (OOP) costs for which they should be exempt. To date, no work has assessed how gaps in enforcing the ACA’s cost-sharing exemption affect patients with chronic conditions, who have higher OOP costs overall, which increases the financial burden from their health care.

**Objective:**

To determine the relative incidence, magnitude, and determinants of cost-sharing for preventive care among individuals with chronic conditions compared with individuals without such conditions.

**Design, Setting, and Participants:**

This cohort study used propensity score matching of patients insured through their employers or the ACA Marketplaces, using claims and remittance data from Symphony Health Solutions’ Integrated DataVerse from 2017 to 2020. Analysis was completed between November 2024 and November 2025.

**Exposure:**

Presence of ambulatory care–sensitive conditions (ACSCs) compared with no chronic conditions.

**Main Outcomes and Measures:**

Primary outcomes included the incidence and amount of costs levied for preventive care. Secondary outcomes included the incidence of cost-sharing for preventive care specifically due to service code misclassification and visit complexity.

**Results:**

A total of 1 262 414 patients (800 693 female patients [63.42%]; mean [SD] age at the time of visit, 54.46 [12.40] years) received 5 236 253 preventive services over 1 984 354 unique visits. The likelihood of a preventive service resulting in cost-sharing was significantly greater among patients with ACSCs compared with those without ACSCs (17.91% [95% CI, 17.69%-17.95%] vs 15.64% [95% CI, 15.69%-15.95%]; *P* < .001). Propensity score–matched models found that individuals with ACSCs had a 19.20% increase (95% CI, 18.87%-19.18%; *P* < .001) in the probability of facing OOP costs for preventive care, and a 20.69% (95% CI, 19.19%-20.91%; *P* < .001) increase in expected preventive OOP costs overall.

**Conclusion and Relevance:**

These findings suggest that patients with chronic conditions were more likely to experience cost-sharing for preventive care and had greater expected spending overall. Standardizing insurer practices regarding cost-sharing exemptions can improve equitable access to high-value preventive care.

## Key Points

**Question:**

Are patients with chronic conditions equally protected from out-of-pocket (OOP) costs for preventive care under the cost-sharing exemptions mandated by the Patient Protection and Affordable Care Act’s (ACA)?

**Findings:**

In this propensity score–matched cohort study of more than 1 million privately insured US patients, those with ambulatory care–sensitive conditions had a 19.20% higher likelihood of cost-sharing for preventive services and 20.69% greater expected OOP spending compared with patients without any chronic conditions.

**Meaning:**

Patients with chronic conditions faced increased preventive care costs, implying that improved compliance with ACA cost-sharing exemptions may reduce financial burdens and disparities in preventive care access.

## Teaser

This cohort study examines the relative incidence, magnitude, and determinants of cost-sharing for preventive care among individuals with chronic conditions compared with individuals without such conditions.

## Introduction

According to the Patient Protection and Affordable Care Act (ACA) of 2010, Americans enrolled in private health insurance—including employer-sponsored insurance and ACA Marketplace coverage—should be exempt from cost-sharing when receiving appropriate preventive care from an in-network practitioner.^1^ The ACA’s guaranteed issue and community rating provisions further protect those with preexisting conditions by requiring insurers to provide coverage without raising premiums, denying coverage, or restricting benefits on the basis of health status.^2^ These protections aimed to reduce the population-level burden of illness by minimizing access and financial burdens to seeking preventive care.^3^ However, these patients still face out-of-pocket (OOP) costs for preventive care, highlighting issues in the operationalization of these mandates.^4^ Recently, insurers challenged this ACA cost-sharing exemption in *Kennedy, Secretary of Health and Human Services v Braidwood Management, Inc*; however, the US Supreme Court upheld its constitutionality, bringing this issue to the forefront of policy discussions.^5^

Although the ACA led to a decline in the incidence of OOP costs for preventive care,^6,7,8,9^ substantial OOP cost burdens remain.^4^ Despite improved access to preventive services,^10^ individuals with chronic conditions may disproportionately bear unexpected OOP costs from preventive care, as their medical care is more complex than that for patients without chronic conditions and may result in a greater likelihood of experiencing billing or administrative errors. For example, patients with diabetes may be more likely to have their wellness visits coded as a maintenance visit for diabetes care, resulting in inadvertent cost-sharing even for purely preventive services.

Of US adults, 76.4% report at least 1 chronic condition,^11^ a proportion that is projected to increase over the next decade.^12^ Chronic conditions represented the leading cause of mortality in 2022,^11^ and their combined direct and indirect costs in the US are estimated to be $3.7 trillion annually, roughly 19.6% of the country’s gross domestic product.^13^ Preventive care plays a critical role in both early detection and improved management of chronic conditions, particularly ambulatory care–sensitive conditions (ACSCs).^14^ Individuals with chronic conditions are also more likely to seek health care services and preventive care.^15^

These dynamics may place patients with chronic conditions at greater risk of unexpected cost-sharing. Households with an individual with chronic conditions are more likely to incur OOP costs compared with households without an individual with chronic conditions.^16^ These costs tend to increase with the number of chronic condition comorbidities,^16,17,18^ frequently resulting in substantial financial burden and associated medical debt—a growing problem as patients are increasingly exposed to higher costs for care.^19,20^

To date, little evidence has shown whether financial protections such as those included in the ACA mitigate these disparities for patients with chronic conditions. Moreover, evidence on the potential mechanisms underlying patient cost-sharing, including billing and insurer processing errors, is needed to motivate potential policy remedies. For example, incorrectly classifying a preventive visit as an office visit or billing for more complex services than were actually rendered may lead to differences in cost-sharing for patients with chronic conditions compared with those without.

In this cohort study, we assessed the association between having an ACSC and the incidence and economic burden of OOP costs for preventive services among individuals with private insurance in the US, using propensity score matching to balance differences between cohorts. We also assessed the extent to which these associations relied on service code misclassifications or practitioner billing practices.

## Methods

### Research Design and Study Sample

This cohort study was approved as exempt—including from informed consent—by the institutional review board at the University of Toronto. This study followed the Strengthening the Reporting of Observational Studies in Epidemiology (STROBE) reporting guideline.^21^

We conducted a retrospective cohort study using the Symphony Health medical claims data between June 2017 and June 2020. The Symphony data provide a large convenience sample of claims for Americans aged 18 to 64 years with employer-sponsored insurance (fully insured and self-insured) and ACA Marketplace plans across all 50 US states and the District of Columbia.^22,23^ Previous work has used these data to study patient outcomes and health equity given its large sample size and rich demographic linkages.^24,25,26^

Our analytical sample included all outpatient visits with preventive services administered, identified on the basis of services recommended by the US Preventive Services Task Force and Centers for Disease Control and Prevention’s Advisory Committee on Immunization Practices (eTable 1 in Supplement 1). We excluded visits not in an outpatient setting, those missing service-level cost data, or patients with a chronic condition diagnosis code not corresponding to an ACSC. We required that patients be continuously enrolled in private insurance for at least 12 months prior to the preventive care visit to ensure that ASCs could be appropriately detected.

Demographic data in the Symphony Health Solutions included self-reported information on household income, education, and race and ethnicity, compiled from sources such as purchase transactions and voter registration data and linked to claims. Where applicable, ethnicity is enhanced using an algorithm leveraging patient name and geographic location. Race and ethnicity data were limited at the point of collection to the following fields: Asian, Hispanic, non-Hispanic Black, non-Hispanic White, and other (not listed above). Demographic data were included for roughly 70% of observed enrollees. Data on race and ethnicity are included because previous work^25^ has found differences in denial rates for preventive care across demographic groups, including race and ethnicity; therefore, we adjusted for these covariates.

The analytical sample was divided into cohorts of patients with and without chronic conditions. Chronic conditions were identified on the basis of the presence of selected ACSCs in the billed diagnosis codes at the time of the preventive visit (eTable 2 in Supplement 1). ACSCs are health conditions for which appropriate primary care delivered through prevention, control, and management can reduce the risk of avoidable hospitalizations.^27^ The nonchronic cohort included all individuals without chronic diagnosis codes present at the time of the preventive visit; patients with chronic conditions listed in the Agency for Healthcare Research and Quality’s Chronic Condition Indicator for *International Statistical Classification of Diseases, Tenth Revision, Clinical Modification* were excluded (eTable 11 in Supplement 2). These included chronic mental health disorders, arthritis, and other conditions not typically managed through preventive care.

### Study Outcomes and Methods

Primary outcomes included the incidence of OOP costs for preventive care and continuous measures for OOP spending in dollars. Subgroup analyses were performed by preventive service category. We excluded all preventive services related to a patient’s diagnosed chronic conditions to rule out measurement error from nonpreventive routine monitoring care. We also measured potential billing code misclassification with an indicator flagging visits where a preventive service was not billed as the primary reason for the visit. To assess whether the classification of a preventive visit as more intensive increased the rate of cost-sharing, we measured billing complexity using the claim’s procedure codes (eTable 3 in Supplement 1). Low-complexity wellness visits are typically billed as preventive care,^28^ whereas high-complexity wellness visits may be mistakenly classified as office visits,^29^ potentially leading to differences in cost-sharing rates. Monetary outcomes were reported in 2020 US dollars.

### Statistical Analysis

We applied 1:1 propensity score matching with replacement to improve covariate balance between cohorts. This ensures that comparisons across groups in cost-sharing for preventive care are not biased by underlying differences in observable patient composition. Propensity scores were calculated using a logistic regression of chronic condition status on patient age, sex, annual household income, education, race and ethnicity, and zip code. All analyses were performed on the matched sample.

Raw differences in outcomes across cohorts were assessed using 2-proportion *z* tests and Mann-Whitney *U* tests, with Cohen *h* used to evaluate effect size of the incidence estimates. After matching, differences in both incidence and monetary burdens across cohorts were assessed using 2-sided *t* tests and logistic and Poisson regression adjustment, respectively. Differences in billing complexity and misclassification were also assessed using logistic regressions. Regression models included fixed effects for health insurer, geography, year, and relative week of year; coefficients were converted to marginal effects at the mean (MEM) for interpretability.^30^ A significance level of *P* < .05 was used for all tests. Analysis was performed using Python statistical software version 3.12.4 (Python Software Foundation) between November 2024 and May 2025.

## Results

The sample consisted of 5 236 253 preventive services delivered to 1 262 414 patients over 1 984 354 unique visits (Table 1). The mean (SD) age of patients receiving care was 54.46 (12.40) years, with 800 693 patients (63.42%) being female and 461 721 (36.8%) being male (eTable 4 in Supplement 1). Overall, 10 857 patients (0.86%) self-identified as Asian, compared with 69 938 (5.54%) Hispanic, 112 860 (8.94%) non-Hispanic Black, 715 789 (56.70%) non-Hispanic White, and 13 120 (1.04%) from racial and ethnic groups unidentified by the former groups. Cohorts were balanced conditional upon the observable covariates after propensity score matching.

**Table 1.** Summary Statistics for Patients With Out-of-Pocket Costs for Preventive Care^a^

Characteristic	Preventive care visits, No. (%) (N = 1 984 354)	Covariate distribution before and after propensity score matching, mean (SD), %					
		Unmatched sample			Matched sample		
		Chronic (n = 3 179 928)	Nonchronic (n = 2 056 325)	SMD	Chronic (n = 1 968 739)	Nonchronic (n = 1 968 739)	SMD
Age, mean (SD), y^b^	53.06 (0.01)	55.40 (12.01)	50.93 (13.22)	0.2635	52.08 (12.32)	52.26 (11.87)	−0.0146
Sex							
Male	552 289 (27.83)	34.95 (47.68)	26.93 (44.36)	0.1742	28.75 (45.26)	27.43 (44.61)	0.0296
Female	1 432 065 (72.17)	65.05 (47.68)	73.07 (44.36)	−0.1742	71.25 (45.26)	72.57 (44.61)	−0.0296
Race and ethnicity							
Asian	16 390 (0.83)	0.85 (9.19)	0.87 (9.28)	−0.0020	0.85 (9.20)	0.89 (9.38)	−0.0035
Hispanic	105 083 (5.30)	5.41 (22.62)	4.83 (21.45)	0.0261	4.80 (21.38)	4.88 (21.55)	−0.0036
Non-Hispanic Black	176 442 (8.89)	9.40 (29.19)	7.93 (27.01)	0.0525	8.17 (27.40)	8.08 (27.25)	0.0034
Non-Hispanic White	1 095 717 (55.22)	56.6 (49.56)	,54.79 (49.77)	0.0365	56.28 (49.60)	55.85 (49.66)	0.0086
Other^c^	19 891 (1.00)	0.99 (9.92)	0.97 (9.78)	0.0028	0.98 (9.83)	0.98 (9.86)	−0.0006
Missing	570 831 (28.77)	26.74 (44.26)	30.61 (46.09)	−0.0857	28.92 (45.34)	29.32 (45.52)	−0.0089
Annual household income, $							
<30 000	248 514 (12.52)	12.90 (33.52)	10.94 (31.22)	0.0603	11.34 (31.70)	10.95 (31.22)	0.0124
30 000-49 999	198 341 (10.00)	10.37 (30.49)	8.82 (28.36)	0.0527	9.27 (29.00)	8.97 (28.57)	0.0104
50 000-74 999	266 479 (13.43)	13.99 (34.69)	12.21 (32.74)	0.0529	12.18 (32.70)	12.53 (33.10)	−0.0106
75 000-99 999	254 915 (12.85)	13.23 (33.88)	12.59 (33.17)	0.0192	13.04 (33.67)	12.88 (33.50)	0.0047
≥100 000	465 888 (23.48)	23.82 (42.60)	25.87 (43.79)	−0.0474	26.36 (44.06)	26.42 (44.09)	−0.0015
Missing	550 217 (27.73)	25.69 (43.69)	29.57 (45.64)	−0.0870	27.83 (44.81)	28.26 (45.03)	−0.0096
Education							
High school or less	377 865 (19.04)	19.50 (39.62)	16.57 (37.19)	0.0761	17.56 (38.05)	16.88 (37.45)	0.0183
Some college	652 374 (32.88)	33.62 (47.24)	31.71 (46.54)	0.0406	31.91 (46.61)	32.33 (46.77)	−0.0089
Associate’s degree or higher	386 227 (19.46)	20.29 (40.22)	21.30 (40.92)	−0.0248	21.74 (41.24)	21.67 (41.20)	0.0015
Missing	567 888 (28.62)	26.60 (44.18)	30.42 (46.01)	−0.0847	28.78 (45.28)	29.12 (45.43)	−0.0072

Abbreviation: SMD, standardized mean difference.

^a^ Propensity score matching regression also adjusted for zip code dummies. Sample sizes for patients with chronic or nonchronic conditions reflect the total number of preventive services received. Descriptive statistics are presented at the unit of observation (the service level) rather than by individual enrollee counts.

^b^ The analytic sample contains individuals aged 18 to 64 years.

^c^ Other race and ethnicity includes any patient not in the listed primary categories.

Before matching, the proportion of preventive services resulting in cost-sharing was significantly greater in patients with chronic conditions compared with those without such conditions (17.91% [95% CI, 17.69%-17.95%] vs 15.64% [95% CI, 15.69%-15.95%]; *P* < .001) (Figure, panel A). Among patients incurring OOP costs, those without chronic conditions faced a greater mean cost per preventive service compared with those with chronic conditions ($61.98 [95% CI, $61.20-$62.75] vs $42.60 [95% CI, $42.12-$43.08]; *P* < .001) (Figure, panel B). The mean visit-level OOP cost for preventive care was also greater for patients without chronic conditions compared with patients with chronic conditions ($87.93 [95% CI, $85.97-$89.88] vs $61.16 [95% CI, $59.90-$62.42]; *P* < .001) (Figure, panel C).

**Figure.**
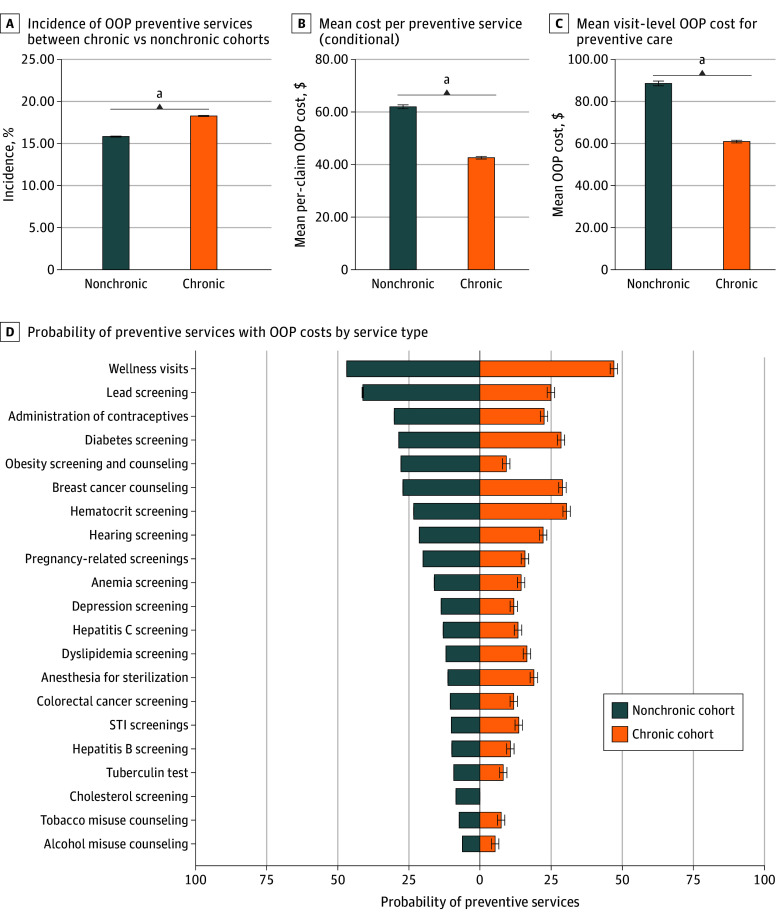
Out-of-Pocket (OOP) Costs for Preventive Services Graphs show incidence of OOP preventive services between patients with chronic vs nonchronic conditions (A), mean cost per preventive service (B), mean visit-level OOP cost for preventive care (C), and probability of preventive services with OOP cost by service type (D). Error bars denote 95% CIs. STI indicates sexually transmitted infection. ^a^*P* < .001.

Patients without chronic conditions exhibited a higher median and wider variance in preventive care cost-sharing compared with those with chronic conditions (eTable 5 in Supplement 1). Distributional differences were similar when including patients without OOP costs (eFigure 1 and eTable 6 in Supplement 1). Differences in the incidence of OOP costs for individuals with chronic conditions were significant across a majority of service types, with wellness visits showing the highest likelihood (Figure, panel D). These differences were also apparent in OOP cost burden and per-capita OOP cost burden, particularly for colorectal cancer screening, wellness visits, diabetes screening, and noninfluenza immunizations (eFigures 2 and 3 in Supplement 1). Finally, we identified significant differences in the likelihood of cost-sharing for those with chronic conditions across a majority of US states (eFigure 4 in Supplement 1).

After matching and regression adjustment, those with chronic conditions had a 3.01 percentage point (MEM, 0.030; 95% CI, 0.029 to 0.031; *P* < .001) or 19.20% increase (95% CI, 18.87%-19.18%; *P* < .001) in the probability of incurring cost-sharing for preventive care compared with those without a chronic condition (Table 2; eTable 7 in Supplement 1). Conditional on incurring costs, those with chronic conditions incurred $1.35 (95% CI, −$1.51 to −$1.19; *P* < .001) less per visit. These patterns were consistently observed across ACSCs (eTable 8 in Supplement 1), and were robust to excluding 2020 from analysis (eTable 9 in Supplement 1). We estimated expected annual patient cost-sharing to be $17.55 per year for patients with chronic conditions (95% CI, $17.33-$17.58 per year) and $14.54 per year (95% CI, $14.49-$14.75 per year) for those without. That is, patients with chronic conditions pay 20.69% (95% CI, 19.19%-20.91%; *P* < .001) more for preventive care alone.

**Table 2.** Likelihood and Monetary Difference in OOP Costs for Preventive Care on Matched Sample, Chronic vs Nonchronic (Conditional on Incurring an OOP Cost)

Characteristic	Model 1^a^^,^^b^		Model 2^a^^,^^c^	
	Logistic regression, MEM (95% CI) (n = 3 760 028)	*P* value	Poisson regression, MEM (95% CI) (n = 3 760 028)	*P* value
Nonchronic vs chronic	0.0301 (0.029-0.031)	<.001	−1.3478 (−1.507 to −1.189)	<.001
Age^d^	−0.0012 (−0.001 to −0.001)	<.001	−0.0641 (−0.071 to −0.058)	<.001
Sex				
Male	−0.0081 (−0.009 to −0.007)	<.001	1.2371 (1.072 to 1.402)	<.001
Female	0 [Reference]		0 [Reference]	
Annual household income, $				
<30 000	−0.0013 (−0.003 to 0.000)	.09	−1.4402 (−1.762 to −1.118)	<.001
30 000-49 999	0.0035 (0.002 to 0.005)	<.001	−1.0305 (−1.294 to −0.767)	<.001
50 000-74 999	0.0071 (0.006 to −0.008)	<.001	−0.5822 (−0.945 to −0.219)	.002
75 000-99 999	0.0033 (0.002 to 0.005)	<.001	−0.8332 (−1.054 to −0.612)	<.001
≥100 000	0 [Reference]	NA	0 [Reference]	NA
Education				
High school or less	0.0168 (0.015 to 0.018)	<.001	0.4821 (0.097 to 0.867)	.01
Some college	0.0096 (0.008 to 0.011)	<.001	0.1708 (−0.020 to 0.362)	.08
Associate’s degree or higher	0 [Reference]	NA	0 [Reference]	NA
Race and ethnicity				
Asian	−0.0114 (−0.016 to −0.007)	<.001	−0.9734 (−1.527 to −0.419)	.001
Hispanic	−0.0045 (−0.006 to −0.003)	<.001	−0.7245 (−1.087 to −0.362)	<.001
Non-Hispanic Black	−0.0007 (−0.002 to 0.001)	.33	−0.5651 (−0.877 to −0.253)	<.001
Non-Hispanic White	0 [Reference]	NA	0 [Reference]	NA
Other^e^	0.0060 (0.002 to 0.010)	.002	0.3302 (−0.214 to 0.874)	.23

Abbreviations: MEM, marginal effect at the mean; NA, not applicable; OOP, out of pocket.

^a^ Coefficient estimates are derived from propensity score matching–adjusted models and reflect MEMs.

^b^ The outcome for model 1 is an indicator for whether any OOP was incurred at the service level.

^c^ The outcome for model 2 is a continuous measure of patient OOP incurred, unconditional on OOP greater than 0.

^d^ The analytic sample contains individuals aged 18 to 64 years.

^e^ Other race and ethnicity includes any patient not in the listed primary categories.

Patients with chronic conditions were 15.1 percentage points (MEM, 0.151; 95% CI, 0.149-0.154; *P* < .001) more likely to have their preventive visit classified as a nonpreventive visit exclusively on the basis of primary diagnosis codes (Table 3; eFigure 5 in Supplement 1). After adjusting for potential misclassifications, patients with chronic conditions had a 3.39 percentage point (MEM, 0.034; 95% CI 0.033-0.035; *P* < .001) increase in the probability of facing cost-sharing, with a nonsignificant difference vs the baseline model.

**Table 3.** Likelihood of Preventive Code Misclassification Between Chronic vs Nonchronic Cohorts on Matched Sample (Conditional on Incurring OOP Costs)

Characteristics	Logistic regression^a^			
	Model 1^b^		Model 2^c^	
	Likelihood of preventive code misclassification, MEM (95% CI) (n = 664 526)	*P* value	Likelihood of OOP cost for preventive care, MEM (95% CI) (n = 3 789 871)	*P* value
Preventive code misclassification	NA	NA	−0.0162 (−0.017 to −0.015)	<.001
Nonchronic vs chronic	0.1514 (0.149 to 0.154)	<.001	0.0339 (0.033 to 0.035)	<.001
Age^d^	0.0028 (0.003 to 0.003)	<.001	−0.0012 (−0.001 to −0.001)	<.001
Sex				
Male	0.1180 (0.115 to 0.121)	<.001	−0.0074 (−0.008 to −0.007)	<.001
Female	0 [Reference]		0 [Reference]	
Annual household income, $				
<30 000	0.0235 (0.019 to 0.028)	<.001	−0.0002 (−0.002 to 0.001)	.76
30 000-49 999	0.0214 (0.017 to 0.026)	<.001	0.0048 (0.003 to 0.006)	<.001
50 000-74 999	0.0109 (0.007 to 0.015)	<.001	0.0081 (0.007 to 0.009)	<.001
75 000-99 999	0.0062 (0.002 to 0.010)	.004	0.0039 (0.003 to 0.005)	<.001
≥100 000	0 [Reference]	NA	0 [Reference]	NA
Education				
High school or less	0.0044 (−5.15 × 10^5^ to 0.009)	.05	0.0175 (0.016 to 0.019)	<.001
Some college	−0.0011 (−0.005 to 0.003)	.57	0.0099 (0.009 to 0.011)	<.001
Associate’s degree or higher	0 [Reference]	NA	0 [Reference]	NA
Race and ethnicity				
Asian	0.0059 (−0.008 to 0.020)	.40	−0.0131 (−0.018 to −0.009)	<.001
Hispanic	0.0084 (0.002 to 0.014)	.005	−0.0046 (−0.007 to −0.003)	<.001
Non-Hispanic Black	0.0022 (−0.002 to 0.007)	.35	−0.0004 (−0.002 to 0.001)	.60
Non-Hispanic White	0 [Reference]	NA	0 [Reference]	NA
Other^e^	−0.0081 (−0.020 to 0.004)	.18	0.0072 (0.003 to 0.011)	<.001

Abbreviations: MEM, marginal effect at the mean; NA, not applicable; OOP, out of pocket.

^a^ Coefficient estimates are derived from propensity score matching–adjusted models and reflect MEMs.

^b^ The outcome for model 1 is an indicator for whether the preventive service was not classified as preventive in primary diagnosis.

^c^ The outcome for model 2 is an indicator for whether any OOP was incurred at the service level.

^d^ The analytic sample contains individuals aged 18 to 64 years.

^e^ Other race and ethnicity includes any patient not in the listed primary categories.

Among individuals receiving wellness visits, individuals with chronic conditions had a 9.10 percentage point (MEM, 0.09; 95% CI 0.09-0.10; *P* < .001) increase in the probability of having their wellness visit billed as high-complexity (Table 4; eTable 10 in Supplement 1). Moreover, individuals receiving high-complexity wellness visits had a 100.64% (MEM, 1.0064; 95% CI, 0.93-1.09; *P* < .001) increase in the probability of incurring cost-sharing for preventive care, compared with those with a low-complexity wellness visit.

**Table 4.** Likelihood of OOP Cost-Sharing Between High-Complexity vs Low-Complexity Wellness Check on Matched Sample (Conditional on Incurring an OOP Cost)

Characteristic	Logistic regression^a^			
	Model 1, high-complexity wellness check, MEM (95% CI) (n = 82 098)^b^	*P* value	Model 2, incurred OOP cost, MEM (95% CI) (n = 22 522)^c^	*P* value
High-complexity vs low-complexity wellness check	NA	NA	1.0064 (0.926 to 1.087)	<.001
Chronic vs nonchronic	0.0009 (0.000 to 0.001)	<.001	−0.0060 (−0.020 to 0.008)	.40
Age^d^	0.0002 (0.000 to 0.000)	<.001	−0.0038 (−0.004 to-0.003)	<.001
Sex				
Male	−0.0004 (−0.001 to-0.000)	<.001	0.0027 (−0.012 to 0.017)	.71
Female	0 [Reference]		0 [Reference]	
Annual household income, $				
<30 000	0.0004 (−0.000 to 0.001)	.20	−0.0914 (−0.119 to −0.064)	<.001
30 000-49 999	0.0005 (−0.000 to 0.001)	.19	−0.0487 (−0.077 to-0.021)	.001
50 000-74 999	0.0005 (−0.000 to 0.001)	.68	−0.0427 (−0.068 to-0.018)	.001
75 000-99 999	−0.0002 (−0.001 to 0.000)	.47	−0.0049 (−0.030 to0.020)	.65
≥100 000	0 [Reference]	NA	0 [Reference]	NA
Education				
High school or less	5.225 × 10^5^ (−0.001 to 0.001)	.87	−0.0274 (−0.053 to-0.002)	.04
Some college	−3.689 × 10^5^ (−0.001 to 0.000)	.83	−0.0166 (−0.038 to 0.005)	.13
Associate’s degree or higher	0 [Reference]	NA	0 [Reference]	NA
Race and ethnicity				
Asian	0.0007 (−0.001 to 0.003)	.50	−0.0473 (−0.133 to 0.038)	.28
Hispanic	0.001 (6.32 × 10^5^ to 0.002)	.04	−0.0617 (−0.092 to-0.032)	<.001
Non-Hispanic Black	−0.0002 (−0.001 to 0.000)	.63	0.0085 (−0.018 to 0.035)	.52
Non-Hispanic White	0 [Reference]	NA	0 [Reference]	NA
Other^e^	−0.0005 (−0.002 to 0.001)	.40	−0.0564 (−0.121 to 0.009)	.09

Abbreviations: MEM, marginal effect at the mean; NA, not applicable; OOP, out of pocket.

^a^ Coefficient estimates are derived from propensity score matching–adjusted models and reflect marginal effects at the mean.

^b^ The outcome for model 1 is an indicator for whether the wellness check was coded as high complexity.

^c^ The outcome for model 2 is an indicator for whether any OOP was incurred at the service level.

^d^ The analytic sample contains individuals age 18 to 64 years.

^e^ Other race and ethnicity includes any patient not in the listed primary categories.

## Discussion

In this cohort study, we examined the association between certain chronic conditions and patient cost-sharing for preventive care among patients for whom preventive care is ostensibly cost-sharing exempt by the ACA. This cost-sharing exemption is one of the most popular provisions of the ACA.^24,26^ The Supreme Court recently upheld its constitutionality, ensuring continued access to free preventive care and reinforcing the policy relevance of our findings.^5^

We found that individuals with chronic conditions—measured using ACSCs—had a 3.01 percentage point increase in the probability of incurring OOP cost for preventive care. This corresponded to a 19.20% increase in the likelihood of facing cost-sharing. These results were consistently observed in both the matched and unmatched sample, suggesting that they are not an artifact of our matching approach or associated with compositional differences across cohorts.

Conditional on facing cost-sharing, we estimate that patients with chronic conditions incur slightly lower costs for preventive care. However, according to the mean rates of preventive care utilization and incidence of cost-sharing (Figure), we estimate expected annual patient cost-sharing to be $17.55 per year for patients with chronic conditions and $14.54 per year for those without. That is, patients with chronic conditions expect to pay 20.69% more for preventive care alone. Even wider disparities may emerge at the upper end of the OOP cost distribution, given the wide variance in OOP cost burden for preventive care (Table 3; eTable 6 in Supplement 1).

OOP costs have been shown to reduce the demand for preventive service utilization, raising concerns about the OOP burden faced by those with chronic conditions.^31^ Properly enforcing the cost-sharing exemption and reducing these financial barriers could encourage individuals—particularly from vulnerable populations—to continue seeking preventive care.^32^

These disparities are not associated with either preventive services related to a specific chronic condition or billing code misclassification. Our analysis excluded preventive services related to a patient’s ACSC; in addition, our measure of billing errors was conservative and included any visit where preventive care was administered despite the overall visit not being classified as preventive. Even after adjusting for these potential errors, the association between chronic condition status and cost-sharing was not statistically significantly different from the unadjusted estimate.

Individuals with chronic conditions who received wellness visits were more likely to have their visits coded as complex, potentially causing a disconnect between physician billing and insurer processing of a claim. Hence, one possible mechanism underlying the estimated results is that physicians may code preventive care for patients with chronic conditions as inherently more complex, leading insurers to process a visit as nonpreventive and issue cost-sharing.

Numerous mechanisms could mediate the observed associations between cohorts, cost-sharing, and billing errors or visit complexities. Even after conditioning on potential visit misclassification, physicians may not appropriately leverage modifier codes to distinguish preventive services from problem-oriented ones.^33^ Although preventive visits cover management of minor problems that do not require the performance of evaluation and management services (ie, history, examination, and medical decision-making), physicians may unknowingly provide services beyond the scope of preventive care coverage.^33^

Our results, therefore, have important policy implications for ensuring broad access to preventive care. First, as best practice, preventive service codes should be listed as the primary reason of the visit, appended with billing modifier code for preventive care (ie, 33) to avoid unexpected OOP bills.^34^ Second, preventive care visits involving the diagnosis of chronic conditions requiring substantial evaluation and management services should append the billing modifier code (ie, 25) to distinguish between office-oriented service and preventive services to prevent unintended cost-sharing. Finally, standardized guidelines for preventive care—perhaps including explicit guidance for coverage of patients with and without chronic conditions—may prevent inappropriate billing of patients. In the current absence of such standardization, billing requirements for cost-sharing exemption vary widely across insurers. Without carefully considering this complexity, vulnerable patients end up paying more for high-value care.

### Limitations

This study was not without limitations. First, we could not directly control for differences in practitioner billing, as practitioners could not be consistently identified across insurers in our data. We are able to adjust for insurer fixed effects; however, if patients with chronic conditions see different practitioners with idiosyncratic differences in billing practices, this could affect our estimated relationships. Additionally, our results may reflect differences across patients who enroll in health insurance plans with different policies for exempting preventive services from cost-sharing (eg, plans with different billing requirements to classify a service as preventive). Although cost-sharing exemptions for preventive services are not uniform across insurers, they typically do not differ across insurance plans; hence, this concern is likely addressed by our fixed effects.

Second, there is potential misclassification of patients with chronic conditions as belonging to the nonchronic cohort given our reliance on diagnosis codes in defining our cohorts.^35,36,37^ Any bias from this misclassification is likely minimal, as practitioners have financial incentives to include diagnosis codes for ACSCs even when providing preventive care; we also required 12 months of continuous enrollment for patients to minimize incorrectly assigning patients to the nonchronic cohort. This misclassification, if it persists, would generate bias serving only to attenuate our estimated associations.

Third, related to this, our analytical sample was constructed at the visit level, and hence our findings reflect cost-sharing experiences for those who take up preventive care, rather than the broader insured population. Conditioning on utilization may bias our results if individuals with chronic conditions systematically differ from other patients in their likelihood of seeking preventive care. To address this, we report an aggregate measure of expected costs for preventive care taking into account the relative frequency of seeking care at the patient level; these results suggest that patients with chronic conditions still experience higher overall rates of cost-sharing even after taking this selection into account.

Fourth, although our data provided rich demographic information, this information was incomplete for some patients in our sample; estimates of heterogeneity in effect sizes may be understated if missing demographic information is correlated with patients in more marginalized situations (eg, those without a valid credit score or voter registration). The Symphony data provide broad geographic coverage and rich demographic linkages, but do not include identifiers for self-insured patients and are not fully representative of all privately insured adults as a convenience sample.

Fifth, our estimates compare patients without chronic conditions with those with ACSCs only. These conditions represent a large fraction of patients with chronic conditions, and management for these conditions relies heavily on preventive primary care. Hence, improving cost-sharing–exempt access to preventive care for patients with these conditions is particularly relevant. However, we excluded patients with other chronic conditions (eg, arthritis or mental health conditions) from our analysis; our results therefore do not generalize to all US patients with chronic conditions.

## Conclusions

This cohort study examined the associations between a patient’s chronic conditions and potentially unexpected cost-sharing for preventive care, as well as the potential mechanisms underlying these bills. Patients with ACSCs were significantly more likely to incur cost-sharing and had higher expected spending on preventive care. Evidence from this study reinforces the need for federal guidance to standardize coding guidelines for cost-sharing exempt preventive services, to ensure that health care legislation such as the ACA is operationalized as intended.

## Notes

Supplement 1. eTable 1. List of Procedure and Diagnostic Codes Used to Identify Preventive Claims eTable 2. *ICD-10* Diagnosis Codes for Ambulatory Care Sensitive Conditions eTable 3. *CPT* Procedure Codes for Wellness Visit Based on Complexity eTable 4. Summary Statistics for Patients with OOP Costs for Preventive Care, Patient Level eTable 5. Distribution of Per Capita and Per Visit OOP Costs for Preventive Services Among Patients with Chronic Conditions vs. Non-Chronic Conditions (Conditional on Incurring an OOP Cost) eFigure 1. Magnitude of Unexpected OOP Cost for Preventive Services Between Individuals with Chronic Conditions vs. Non-chronic Conditions eTable 6. Distribution of Per Capita and Per Visit OOP Unexpected Cost for Preventive Services Among Patients with Chronic Conditions vs. Non-chronic Conditions (Unconditional on Incurring an OOP Cost) eFigure 2. Aggregate Unexpected OOP Cost Burden by Preventive Service Type Between Individuals with Chronic Conditions vs. Non-chronic Conditions eFigure 3. Per-capita Unexpected OOP Cost Burden by Preventive Service Type Between Individuals with Chronic Conditions vs. Non-chronic Conditions eFigure 4. Differences in Percentage of Preventive Services Incurring an Unexpected OOP Cost for Preventive Care Between Individuals with Chronic Conditions vs. Non-Chronic Conditions eTable 7. Likelihood and Monetary Difference in Unexpected OOP Costs for Preventive Care on Unmatched Sample, Chronic versus Non-chronic (Conditional on Incurring an OOP Cost) eTable 8. Likelihood and Monetary Difference in OOP Cost for Preventive Care on Matched Sample, Selected Ambulatory Sensitive Conditions (Asthma and Diabetes) vs. Non-chronic Conditions (Conditional on Incurring an OOP Cost) eTable 9. Likelihood and Monetary Difference in OOP Costs for Preventive Care on Matched Sample, Chronic versus Non-chronic (Years 2017-2019) eFigure 5. Percentage Distribution of Preventive Service Classification Between Individuals with Chronic Condition vs. Non-chronic Condition eTable 10. Likelihood of Unexpected OOP cost Between High- vs. Low-Complexity Wellness Check on Unmatched Sample (Conditional on Incurring an OOP Cost)

Supplement 2. eTable 11. Chronic Condition Indicator Refined (CCIR) for* ICD-10-CM*

Supplement 3. Data Sharing Statement
