# Supplementary material for: Role of Chronic Conditions in Out-of-Pocket Costs for Preventive Care in the US
Source: JAMA Netw Open. 2026 Jan 8;9(1):e2553157. doi: 10.1001/jamanetworkopen.2025.53157 (PMC12784232; doi:10.1001/jamanetworkopen.2025.53157)
Supplement: Supplement 1. — eTable 1. List of Procedure and Diagnostic Codes Used to Identify Preventive Claims eTable 2. ICD-10 Diagnosis Codes for Ambulatory Care Sensitive Conditions eTable 3. CPT Procedure Codes for Wellness Visit Based on Complexity eTable 4. Summary Statistics for Patients with OOP Costs for Preventive Care, Patient Level eTable 5. Distribution of Per Capita and Per Visit OOP Costs for Preventive Services Among Patients with Chronic Conditions vs. Non-Chronic Conditions (Conditional on Incurring an OOP Cost) eFigure 1. Magnitude of Unexpected OOP Cost for Preventive Services Between Individuals with Chronic Conditions vs. Non-chronic Conditions eTable 6. Distribution of Per Capita and Per Visit OOP Unexpected Cost for Preventive Services Among Patients with Chronic Conditions vs. Non-chronic Conditions (Unconditional on Incurring an OOP Cost) eFigure 2. Aggregate Unexpected OOP Cost Burden by Preventive Service Type Between Individuals with Chronic Conditions vs. Non-chronic Conditions eFigure 3. Per-capita Unexpected OOP Cost Burden by Preventive Service Type Between Individuals with Chronic Conditions vs. Non-chronic Conditions eFigure 4. Differences in Percentage of Preventive Services Incurring an Unexpected OOP Cost for Preventive Care Between Individuals with Chronic Conditions vs. Non-Chronic Conditions eTable 7. Likelihood and Monetary Difference in Unexpected OOP Costs for Preventive Care on Unmatched Sample, Chronic versus Non-chronic (Conditional on Incurring an OOP Cost) eTable 8. Likelihood and Monetary Difference in OOP Cost for Preventive Care on Matched Sample, Selected Ambulatory Sensitive Conditions (Asthma and Diabetes) vs. Non-chronic Conditions (Conditional on Incurring an OOP Cost) eTable 9. Likelihood and Monetary Difference in OOP Costs for Preventive Care on Matched Sample, Chronic versus Non-chronic (Years 2017-2019) eFigure 5. Percentage Distribution of Preventive Service Classification Between Individuals with Chronic Condition vs. Non-chronic [file jamanetwopen-e2553157-s001.pdf]

Supplemental Online Content

Tran A, Laporte A, Nauenberg E, Hoagland A. Role of chronic conditions in out-of-pocket costs for preventive care in the US. *JAMA Netw Open*. 2026;9(1):e2553157. doi:10.1001/jamanetworkopen.2025.53157

eTable 1. List of Procedure and Diagnostic Codes Used to Identify Preventive Claims
eTable 2. *ICD-10* Diagnosis Codes for Ambulatory Care Sensitive Conditions
eTable 3. *CPT* Procedure Codes for Wellness Visit Based on Complexity
eTable 4. Summary Statistics for Patients with OOP Costs for Preventive Care, Patient Level
eTable 5. Distribution of Per Capita and Per Visit OOP Costs for Preventive Services Among Patients with Chronic Conditions vs. Non-Chronic Conditions (Conditional on Incurring an OOP Cost)
eFigure 1. Magnitude of Unexpected OOP Cost for Preventive Services Between Individuals with Chronic Conditions vs. Non-chronic Conditions
eTable 6. Distribution of Per Capita and Per Visit OOP Unexpected Cost for Preventive Services Among Patients with Chronic Conditions vs. Non-chronic Conditions (Unconditional on Incurring an OOP Cost)
eFigure 2. Aggregate Unexpected OOP Cost Burden by Preventive Service Type Between Individuals with Chronic Conditions vs. Non-chronic Conditions
eFigure 3. Per-capita Unexpected OOP Cost Burden by Preventive Service Type Between Individuals with Chronic Conditions vs. Non-chronic Conditions
eFigure 4. Differences in Percentage of Preventive Services Incurring an Unexpected OOP Cost for Preventive Care Between Individuals with Chronic Conditions vs. Non-Chronic Conditions
eTable 7. Likelihood and Monetary Difference in Unexpected OOP Costs for Preventive Care on Unmatched Sample, Chronic versus Non-chronic (Conditional on Incurring an OOP Cost)
eTable 8. Likelihood and Monetary Difference in OOP Cost for Preventive Care on Matched Sample, Selected Ambulatory Sensitive Conditions (Asthma and Diabetes) vs. Non-chronic Conditions (Conditional on Incurring an OOP Cost)
eTable 9. Likelihood and Monetary Difference in OOP Costs for Preventive Care on Matched Sample, Chronic versus Non-chronic (Years 2017-2019)

© 2026 Tran A et al. *JAMA Network Open*.

eFigure 5. Percentage Distribution of Preventive Service Classification Between Individuals with Chronic Condition vs. Non-chronic Condition
eTable 10. Likelihood of Unexpected OOP cost Between High- vs. Low-Complexity Wellness Check on Unmatched Sample (Conditional on Incurring an OOP Cost)

This supplemental material has been provided by the authors to give readers additional information about their work.

© 2026 Tran A et al. *JAMA Network Open*.

eTable 1: List of Procedure and Diagnostic Codes Used to Identify Preventive Claims

Service Type	*CPT* Procedural Codes	Required Accompanying *ICD-10-CM* Diagnosis Codes
Administration of Contraceptives	96372, 57170, 58340, 58565, 58600, 58605, 58611, 58615, 58670, 58671, 74740, A4261, A4264, A4266, 11976, 11981, 11982, 11983, 58300, 58301, J1050, J1051, J1055, J1056, J7302, J7306, J7307, S4981, S4989, J7300, Q0090	
Alcohol misuse counseling	99401, 99402, 99403, 99404, 99408, 99409, 99411, 99412, G0396, G0397, G0442	Z7189, Z1389, Z7141
Anemia screening	36415, 36416, 85013, 85014, 85018, 85041	Z331, Z3400, Z3401, Z3402, Z3403, Z3480, Z3481, Z3482, Z3483, Z3490, Z3491, Z3492, Z3493, Z36, O0900, O0901, O0902, O0903, O0910, O0911, O0912, O0913, O09211, O09212, O09213, O09219, O09291, O09292, O09293, O09299, O0930, O0931, O0932, O0933, O0940, O0941, O0942, O0943, O09511, O09512, O09513, O09519, O09521, O09522, O09523, O09529, O09611, O09612, O09613, O09619, O09621, O09622, O09623, O09629, O0970, O0971, O0972, O0973, O09811, O09812, O09813, O09819, O09821, O09822, O09823, O09829, O09891, O09892, O09893, O09899, O0990, O0991, O0992, O0993, O3680X0, O3680X1, O3680X2, O3680X3, O3680X4, O3680X5, O3680X9, O30001, O30002, O30003, O30009, O30011, O30012, O30013, O30019, O30021, O30022, O30023, O30031, O30032, O30033, O30039, O30041, O30042, O30043, O30049, O30091, O30092, O30093,

© 2026 Tran A et al. *JAMA Network Open*.

Service Type	*CPT* Procedural Codes	Required Accompanying *ICD-10-CM* Diagnosis Codes
		O30099, O30101, O30102, O30103, O30109, O30111, O30112, O30113, O30119, O30121, O30122, O30123, O30129, O30191, O30192, O30193, O30199, O30201, O30202, O30203, O30209, O30211, O30212, O30213, O30219, O30221, O30222, O30223, O30229, O30291, O30292, O30293, O30299, O30801, O30802, O30803, O30809, O30811, O30812, O30813, O30819, O30821, O30822, O30823, O30829, O30891, O30892, O30893, O30899, O3090, O3091, O3092, O3093
Anesthesia for sterilization	00851, 00940, 00942, 00950, 00952, 01960, 01965, 01966, 01967, 01968	Z302
Bacteriuria screening	81007	Z331, Z3400, Z3401, Z3402, Z3403, Z3480, Z3481, Z3482, Z3483, Z3490, Z3491, Z3492, Z3493, Z36, O0900, O0901, O0902, O0903, O0910, O0911, O0912, O0913, O09211, O09212, O09213, O09219, O09291, O09292, O09293, O09299, O0930, O0931, O0932, O0933, O0940, O0941, O0942, O0943, O09511, O09512, O09513, O09519, O09521, O09522, O09523, O09529, O09611, O09612, O09613, O09619, O09621, O09622, O09623, O09629, O0970, O0971, O0972, O0973, O09811, O09812, O09813, O09819, O09821, O09822, O09823, O09829, O09891, O09892, O09893, O09899, O0990, O0991, O0992, O0993, O3680X0, O3680X1, O3680X2, O3680X3, O3680X4, O3680X5, O3680X9, O30001, O30002, O30003, O30009, O30011, O30012, O30013, O30019, O30021, O30022, O30023, O30031, O30032, O30033, O30039, O30041,

© 2026 Tran A et al. *JAMA Network Open*.

Service Type	*CPT* Procedural Codes	Required Accompanying *ICD-10-CM* Diagnosis Codes
		O30042, O30043, O30049, O30091, O30092, O30093, O30099, O30101, O30102, O30103, O30109, O30111, O30112, O30113, O30119, O30121, O30122, O30123, O30129, O30191, O30192, O30193, O30199, O30201, O30202, O30203, O30209, O30211, O30212, O30213, O30219, O30221, O30222, O30223, O30229, O30291, O30292, O30293, O30299, O30801, O30802, O30803, O30809, O30811, O30812, O30813, O30819, O30821, O30822, O30823, O30829, O30891, O30892, O30893, O30899, O3090, O3091, O3092, O3093
Breast Cancer counseling	99201, 99202, 99203, 99204, 99205, 99211, 99212, 99213, 99214, 99215, 99385, 99386, 99387, 99395, 99396, 99397, 96040, S0265	Z803, Z8041, Z1501, Z1502, Z853, Z8543
Breast Cancer screening	77057, 77052, G0202, 77067, 77065, 77066, 76083, 76092	
Breastfeeding support	99201, 99202, 9203, 99211, 99212, 99213, 99214, 99241,99242, 99243, 99244, 99245, 99341, 99342, 99343, 99344,99345, 99347, 99348, 99349, 99350, 99401, 99402, 99403,99404, 99411, 99412, A4281,A4282, A4283, A4284, A4285, A4286, E0602, E0603, E0604, S9443	Z391
Cervical Cance	88141, 88142, 88143, 88147, 88148, 88150, 88152, 88153, 88154, 88155, 88164, 88165, 88166, 88167, 88174, 88175,	Z0000, Z0001, Z01419, Z124

© 2026 Tran A et al. *JAMA Network Open*.

Service Type	*CPT* Procedural Codes	Required Accompanying *ICD-10-CM* Diagnosis Codes
r screening	G0101, G0123, G0124, G0141, G0143, G0144, G0145, G0147, G0148, P3000, P3001, Q0091	
Cholesterol screening	36415, 36416, 80061, 82465, 83718, 83719, 83721, 84478	Z0000, Z0001, Z13220
Colorectal Cancer screening	44388, 44389, 44392, 44393, 44394, 45300, 45301, 45302, 45303, 45304, 45305, 45306, 45307, 45308, 45309, 45310, 45311, 45312, 45313, 45314, 45315, 45316, 45317, 45318, 45319, 45320, 45330, 45331, 45332, 45333, 45334, 45335, 45338, 45339, 45340, 45378, 45379, 45380, 45381, 45382, 45383, 45384, 45385, 45386, 74263, 82270, 82274, 88304, 88305, G0104, G0105, G0106, G0107, G0120, G0121, G0122, G0328, G0394, S0601, S3890	Z0000, Z0001, Z1210, Z1211, Z1212, Z800, Z8371, Z8379
Depression screening	96127, 96160, 96161, 99420, G0444	Z1389
Developmental/autism screening	96110, G0451	Z00129, Z134
Diabetes screening	36415, 36416, 82947, 82948, 82950, 82951, 82952, 83036	Z0000, Z0001, Z131, I10, I110, I119, I120, I129, I130, I1310, I1311, I132, I150, I151, I152, I158, I159, N262, O10011, O10012, O10013, O10019, O1002, O1003, O10111, O10112, O10113, O10119, O1012, O1013, O10211, O10212, O10213, O10219, O1022, O1023,

© 2026 Tran A et al. *JAMA Network Open*.

Service Type	*CPT* Procedural Codes	Required Accompanying *ICD-10-CM* Diagnosis Codes
		O10311, O10312, O10313, O10319, O1032, O1033, O10411, O10412, O10413, O10419, O1042, O1043, O10911, O10912, O10913, O10919, O1092, O1093, O111, O112, O113, O119, O131, O132, O139, O161, O162, O163, O169
Dyslipidemia screening	36415, 36416, 80061, 82465, 83718, 83719, 83721, 84478	Z00129, Z13220
Hepatitis B screening	36415, 36416, 87340, 87341	Z331, Z3400, Z3401, Z3402, Z3403, Z3480, Z3481, Z3482, Z3483, Z3490, Z3491, Z3492, Z3493, Z36, O0900, O0901, O0902, O0903, O0910, O0911, O0912, O0913, O09211, O09212, O09213, O09219, O09291, O09292, O09293, O09299, O0930, O0931, O0932, O0933, O0940, O0941, O0942, O0943, O09511, O09512, O09513, O09519, O09521, O09522, O09523, O09529, O09611, O09612, O09613, O09619, O09621, O09622, O09623, O09629, O0970, O0971, O0972, O0973, O09811, O09812, O09813, O09819, O09821, O09822, O09823, O09829, O09891, O09892, O09893, O09899, O0990, O0991, O0992, O0993, O3680X0, O3680X1, O3680X2, O3680X3, O3680X4, O3680X5, O3680X9, O30001, O30002, O30003, O30009, O30011, O30012, O30013, O30019, O30021, O30022, O30023, O30031, O30032, O30033, O30039, O30041, O30042, O30043, O30049, O30091, O30092, O30093, O30099, O30101, O30102, O30103, O30109, O30111, O30112, O30113, O30119, O30121, O30122, O30123,

© 2026 Tran A et al. *JAMA Network Open*.

Service Type	*CPT* Procedural Codes	Required Accompanying *ICD-10-CM* Diagnosis Codes
		O30129, O30191, O30192, O30193, O30199, O30201, O30202, O30203, O30209, O30211, O30212, O30213, O30219, O30221, O30222, O30223, O30229, O30291, O30292, O30293, O30299, O30801, O30802, O30803, O30809, O30811, O30812, O30813, O30819, O30821, O30822, O30823, O30829, O30891, O30892, O30893, O30899, O3090, O3091, O3092, O3093
Hepatitis C screening	86803, 86804	Z0000, Z0001, Z00129, Z761, Z762, Z3480, Z3490, Z331, Z333, O0900, O0910, O09A0, O09291, O0940, O09211, O0930, O09521, O09511, O09611, O09621, O09819, O09821, O09822, O09823, O09829, O09891, O09892, O09893, O09899, Z390, O0990, O0991, O0992, O0993, Z391, Z392, Z01411, Z01419
Immunization for influenza	90653, 90654, 90655, 90656, 90657, 90658, 90660, 90661, 90662, 90664, 90666, 90667, 90668, 90672, 90685, 90686, 90687, 90688, Q2033, Q2034, Q2035, Q2036, Q2037, Q2038, Q2039	Z23
Immunizations (non-influenza)	90460, 90461, 90465, 90466, 90467, 90468, 90470, 90471, 90472, 90473, 90474, 90632, 90633, 90634, 90636, 90644, 90645, 90646, 90647, 90648, 90649, 90650, 90669, 90670, 90,680, 90,681, 90,696, 90698, 90700, 90701, 90702, 90703, 90704, 90705, 90706, 90707, 90708, 90710, 90712, 90713, 90714, 90715, 90716, 90718, 90719, 90720, 90721, 90723, 90732, 90733, 90734, 90736, 90740, 90743, 90744, 90746, 90747, 90748, G0008, G0009, G0010, G0377, G9141, J3530, S0195	Z23
Lead screening	36415, 36416, 83655	Z00129, Z77011

© 2026 Tran A et al. *JAMA Network Open*.

Service Type	*CPT* Procedural Codes	Required Accompanying *ICD-10-CM* Diagnosis Codes
Management of contraceptives	V2501, V2502, V2503, V2509, V2511, V2512, V2513, V2540, V2541, V2542, V2543, V2549, V255, V258, V259, Z30011, Z30012, Z30013, Z30014, Z30018, Z30019, Z3009, Z3040, Z3041, Z3042, Z30430, Z30431, Z30432, Z30433, Z3049, Z308, Z309	
Obesity screening and counseling	97802, 97803, 97804, 99401, 99402, 99403, 99404, G0446, G0447, 99411, 99412, G0270, G0271, G0449, S9470	Z6838, Z6839, Z6841, Z6842, Z6843, Z6844, Z6845, E6601, E6609, E661, E668, E66, I110, I119, I120, I129, I130, I1310, I1311, I132, I150, I151, I152, I158, I159, N262, O10011, O10012, O10013, O10019, O1002, O1003, O10111, O10112, O10113, O10119, O1012, O1013, O10211, O10212, O10213, O10219, O1022, O1023, O10311, O10312, O10313, O10319, O1032, O1033, O10411, O10412, O10413, O10419, O1042, O1043, O10911, O10912, O10913, O10919, O1092, O1093, O111, O112, O113, O119, O131, O132, O139, O161, O162, O163, O169, E0800, E0801, E0810, E0811, E0821, E0822, E0829, E08311, E08319, E08321, E08329, E08331, E08339, E08341, E08349, E08351, E08359, E0836, E0839, E0840, E0841, E0842, E0843, E0844, E0849, E0851, E0852, E0859, E08610, E08618, E08620, E08621, E08622, E08628, E08630, E08638, E08641, E08649, E0865, E0869, E088, E089, E0900, E0901, E0910, E0911, E0921, E0922, E0929, E09311, E09319, E09321, E09329, E09331, E09339, E09341, E09349, E09351, E09359, E0936, E0939, E0940, E0941, E0942, E0943, E0944, E0949, E0951, E0952, E0959, E09610, E09618, E09620, E09621, E09622, E09628, E09630, E09638, E09641, E09649, E0965, E0969, E098, E099, E1010, E1011, E1021, E1022, E1029, E10311, E10319, E10321, E10329, E10331, E10339, E10341,

© 2026 Tran A et al. *JAMA Network Open*.

Service Type	*CPT* Procedural Codes	Required Accompanying *ICD-10-CM* Diagnosis Codes
		E10349, E10351, E10359, E1036, E1039, E1040, E1041, E1042, E1043, E1044, E1049, E1051, E1052, E1059, E10610, E10618, E10620, E10621, E10628, E10630, E10638, E10641, E10649, E1065, E1069, E108, E109, E1100, E1101, E1121, E1122, E1129, E11311, E11319, E11321, E11329, E11331, E11339, E11341, E11349, E11351, E11359, E1136, E1139, E1140, E1141, E1142, E1143, E1144, E1149, E1151, E1152, E1159, E11610, E11618, E11620, E11621, E11622, E11628, E11630, E11638, E11641, E11649, E1165, E1169, E118, E119, E1300, E1301, E1310, E1311, E1321, E1322, E1329, E13311, E13319, E13321, E13329, E13331, E13339, E13341, E13349, E13351, E13359, E1336, E1339, E1340, E1341, E1342, E1343, E1344, E1349, E1351, E1352, E1359, E13610, E13618, E13620, E13621, E13622, E13628, E13630, E13638, E13641, E13649, E1365, E1369, E138, E139
Pregnancy-related screenings	36415, 36416, 86900, 86901, 82947, 82948, 82,950, 82951, 82952, 83036	Z331, Z3400, Z3401, Z3402, Z3403, Z3480, Z3481, Z3482, Z3483, Z3490, Z3491, Z3492, Z3493, Z36, O0900, O0901, O0902, O0903, O0910, O0911, O0912, O0913, O09211, O09212, O09213, O09219, O09291, O09292, O09293, O09299, O0930, O0931, O0932, O0933, O0940, O0941, O0942, O0943, O09511, O09512, O09513, O09519, O09521, O09522, O09523, O09529, O09611, O09612, O09613, O09619, O09621, O09622, O09623, O09629, O0970, O0971, O0972, O0973, O09811, O09812, O09813, O09819, O09821, O09822, O09823, O09829, O09891, O09892, O09893, O09899, O0990, O0991, O0992, O0993, O3680X0, O3680X1, O3680X2, O3680X3, O3680X4, O3680X5, O3680X9, O30001, O30002, O30003, O30009,

© 2026 Tran A et al. *JAMA Network Open*.

Service Type	*CPT* Procedural Codes	Required Accompanying *ICD-10-CM* Diagnosis Codes
		O30011, O30012, O30013, O30019, O30021, O30022, O30023, O30031, O30032, O30033, O30039, O30041, O30042, O30043, O30049, O30091, O30092, O30093, O30099, O30101, O30102, O30103, O30109, O30111, O30112, O30113, O30119, O30121, O30122, O30123, O30129, O30191, O30192, O30193, O30199, O30201, O30202, O30203, O30209, O30211, O30212, O30213, O30219, O30221, O30222, O30223, O30229, O30291, O30292, O30293, O30299, O30801, O30802, O30803, O30809, O30811, O30812, O30813, O30819, O30821, O30822, O30823, O30829, O30891, O30892, O30893, O30899, O3090, O3091, O3092, O3093
STI screenings	36415, 36416, 86631, 86632, 87110, 87270, 87320, 87490, 87491, 87492, 87801, 87810, G0450, 87590, 87591, 87592, 87850, G0450, 99401, 99402, 99403, 99404, G0445, 86689, 86701, 86702, 86703, 87390, 87391, G0432, G0433, G0435, S3645, 86592, 86593, 87620, 87621, 87622	Z331, Z3400, Z3401, Z3402, Z3403, Z3480, Z3481, Z3482, Z3483, Z3490, Z3491, Z3492, Z3493, Z36, O0900, O0901, O0902, O0903, O0910, O0911, O0912, O0913, O09211, O09212, O09213, O09219, O09291, O09292, O09293, O09299, O0930, O0931, O0932, O0933, O0940, O0941, O0942, O0943, O09511, O09512, O09513, O09519, O09521, O09522, O09523, O09529, O09611, O09612, O09613, O09619, O09621, O09622, O09623, O09629, O0970, O0971, O0972, O0973, O09811, O09812, O09813, O09819, O09821, O09822, O09823, O09829, O09891, O09892, O09893, O09899, O0990, O0991, O0992, O0993, O3680X0, O3680X1, O3680X2, O3680X3, O3680X4, O3680X5, O3680X9, O30001, O30002, O30003, O30009, O30011, O30012, O30013, O30019, O30021, O30022, O30023, O30031, O30032, O30033, O30039, O30041, O30042, O30043, O30049, O30091, O30092, O30093, O30099, O30101, O30102, O30103, O30109, O30111, O30112, O30113, O30119, O30121, O30122,

© 2026 Tran A et al. *JAMA Network Open*.

Service Type	*CPT* Procedural Codes	Required Accompanying *ICD-10-CM* Diagnosis Codes
		O30123, O30129, O30191, O30192, O30193, O30199, O30201, O30202, O30203, O30209, O30211, O30212, O30213, O30219, O30221, O30222, O30223, O30229, O30291, O30292, O30293, O30299, O30801, O30802, O30803, O30809, O30811, O30812, O30813, O30819, O30821, O30822, O30823, O30829, O30891, O30892, O30893, O30899, O3090, O3091, O3092, O3093, V745, V759, V029, V20, V201, V723, V7231, V7262, V7389, Z226, Z228, Z229, Z114, Z1159, Z119, Z206, Z112, V242, Z0000, Z0001, Z124, V726, V7260, V7381, V762, V7646, V7647, V8402, V8404, Z0149, Z1151
Tobacco misuse counseling	99401, 99402, 99403, 99404, 99406, 99407, C9801, C9802, G0436, G0437, S9075, S9453	
Tuberculin test	86580, 99211	R7611, R7612, Z00129, Z111
Vision screening	99173, 99174	Z23, Z761, Z762, Z00129, Z0000, Z0001, Z0100, Z0101, H5231, H5232, H5203, H5213, H52209, H52229, H52219
Wellness visits	99201, 99202, 99203, 99204, 99205, 99211, 99212, 99213, 99214, 99215, 99381, 99382, 99383, 99384, 99388, 99389, 99390, 99391, 99392, 99393, 99394, 99401, 99402, 99403, 99404, 99411, 99412, 99461, G0101, G0344, G0402, G0438, G0439, G0445, S0610, S0612, S0613	Z0000, Z0001, Z00110, Z00111, Z00129

© 2026 Tran A et al. *JAMA Network Open*.

eTable 2. ***ICD-10*** **Diagnosis Codes for Ambulatory Care Sensitive Conditions**

**Chronic Condition**	***ICD-10*** **Codes**
Asthma	J4520, J4521, J4522, J4530, J4531, J4532, J4540, J4541, J4542, J4550, J4551, J4552, J45901, J45902, J45909, J45991, J45998, J8283
Diabetes	O24011, O24012, O24013, O24019, O2402, O2403, O24111, O24112, O24113, O24119, O2412, O2413, O24311, O24312, O24313, O24319, O2432, O2433, O24410, O24414, O24415, O24419, O24420, O24424, O24425, O24429, O24430, O24434, O24435, O24439, O24811, O24812, O24813, O24819, O2482, O2483, O24911, O24912, O24913, O24919, O2492, O2493, Z131, E0800, E0801, E0810, E0811, E0821, E0822, E0829, E08311, E08319, E08321, E083211, E083212, E083213, E083219, E08329, E083291, E083292, E083293, E083299, E08331, E083311, E083312, E083313, E083319, E08339, E083391, E083392, E083393, E083399, E08341, E083411, E083412, E083413, E083419, E08349, E083491, E083492, E083493, E083499, E08351, E083511, E083512, E083513, E083519, E083521, E083522, E083523, E083529, E08353, E083532, E083533, E083539, E083541, E083542, E083543, E083549, E083551, E083552, E083553, E083559, E08359, E083591, E083592, E083593, E083599, E0836, E0837X1, E0837X2, E0837X3, E0837X9, E0839, E0840, E0841, E0842, E0843, E0844, E0849, E0851, E0852, E0859, E08610, E08618, E08620, E08621, E08622, E08628, E08630, E08638, E08641, E08649, E0865, E0869, E088, E089, E0900, E0901, E0910, E0911, E0921, E0922, E0929, E09311, E09319, E09321, E093211, E093212, E093213, E093219, E09329, E093291, E093292, E093293, E093299, E09331, E093311, E093312, E093313, E093319, E09339, E093391, E093392, E093393, E093399, E09341, E093411, E093412, E093413, E093419, E09349, E093491, E093492, E093493, E093499, E09351, E093511, E093512, E093513, E093519, E093521, E093522, E093523, E093529, E093531, E093532, E093533, E093539, E093541, E093542, E093543, E093549, E093551, E093552, E093553, E093559, E09359, E093591, E093592, E093593, E093599, E0936, E0937X1, E0937X2, E0937X3, E0937X9, E0939, E0940, E0941, E0942, E0943, E0944, E0949, E0951, E0952, E0959, E09610, E09618, E09620, E09621, E09622, E09628, E09630, E09638, E09641, E09649, E0965, E0969, E098, E099, E1010, E1011, E1021, E1022, E1029, E10311, E10319, E10321, E103211, E103212, E103213, E103219, E10329, E103291, E103292, E103293, E103299, E10331, E103311, E103312, E103313, E103319, E10339,

© 2026 Tran A et al. *JAMA Network Open*.

**Chronic Condition**	***ICD-10* Codes**
	E103391, E103392, E103393, E103399, E10341, E103411, E103412, E103413, E103419, E10349, E103491, E103492, E103493, E103499, E10351, E103511, E103512, E103513, E103519, E103521, E103522, E103523, E103529, E103531, E103532, E103533, E103539, E103541, E103542, E103543, E103549, E103551, E103552, E103553, E103559, E10359, E103591, E103592, E103593, E103599, E1036, E1037X1, E1037X2, E1037X3, E1037X9, E1039, E1040, E1041, E1042, E1043, E1044, E1049, E1051, E1052, E1059, E10610, E10618, E10620, E10621, E10622, E10628, E10630, E10638, E10641, E10649, E1065, E1069, E108, E109, E1100, E1101, E1110, E1111, E1121, E1122, E1129, E11311, E11319, E11321, E113211, E113212, E113213, E113219, E11329, E113291, E113292, E113293, E113299, E11331, E113311, E113312, E113313, E113319, E11339, E113391, E113392, E113393, E113399, E11341, E113411, E113412, E113413, E113419, E11349, E113491, E113492, E113493, E113499, E11351, E113511, E113512, E113513, E113519, E113521, E113522, E113523, E113529, E113531, E113532, E113533, E113539, E113541, E113542, E113543, E113549, E113551, E113552, E113553, E113559, E11359, E113591, E113592, E113593, E113599, E1136, E1137X1, E1137X2, E1137X3, E1137X9, E1139, E1140, E1141, E1142, E1143, E1144, E1149, E1151, E1152, E1159, E11610, E11618, E11620, E11621, E11622, E11628, E11630, E11638, E11641, E11649, E1165, E1169, E118, E119, E1300, E1301, E1310, E1311, E1321, E1322, E1329, E13311, E13319, E13321, E133211, E133212, E133213, E133219, E13329, E133291, E133292, E133293, E133299, E13331, E133311, E133312, E133313, E133319, E13339, E133391, E133392, E133393, E133399, E13341, E133411, E133412, E133413, E133419, E13349, E133491, E133492, E133493, E133499, E13351, E133511, E133512, E133513, E133519, E133521, E133522, E133523, E133529, E133531, E133532, E133533, E133539, E133541, E133542, E133543, E133549, E133551, E133552, E133553, E133559, E13359, E133591, E133592, E133593, E133599, E1336, E1337X1, E1337X2, E1337X3, E1337X9, E1339, E1340, E1341, E1342, E1343, E1344, E1349, E1351, E1352, E1359, E13610, E13618, E13620, E13621E13622, E13628, E13630, E13638, E13641, E13649, E1365, E1369, E138, E139
Chronic Obstructive Pulmonary Disease (COPD)	J440, J441, J4489, J449

Abbreviation: *ICD, International Classification of Disease*.

© 2026 Tran A et al. *JAMA Network Open*.

eTable 3. *CPT* Procedure Codes for Wellness Visit Based on Complexity

Category	Billing Codes		
		CPT Codes	
	ICD-10	**Low-Complexity**	**High-Complexity**
Wellness Visits	Z0000, Z0001, Z00110, Z00111, Z00129	99381, 99382, 99383, 99384, 99385, 99386, 99387, 99391, 99392, 99393, 99394, 99395, 99396, 99397	99204, 99205, 99214, 99215

Abbreviations: *ICD, International Classification of Disease; CPT, Common Procedural Terminology.*

© 2026 Tran A et al. *JAMA Network Open*.

eTable 4. Summary Statistics for Patients with OOP Costs for Preventive Care, Patient Level

Characteristics	Covariate Distribution Before and After Propensity Score Matching, mean % (SD)					
	Unmatched Sample			Matched Sample		
	Chronic N = 849 704	Non-chronic N = 412 710	SMD	Chronic N = 688 423	Non-chronic N = 415 104	SMD
Patients						
Age[a], mean (SD), y	54.46 (12.40)	49.06 (14.01)	0.4085	52.92 (12.49)	51.36 (12.57)	0.1244
Sex						
Male	36.58 (48.16)	24.00 (42.71)	0.2762	33.10 (47.06)	25.80 (43.76)	0.1605
Female	63.42 (48.16)	76.00 (42.71)	-0.2762	66.90 (47.06)	74.20 (43.76)	-0.1605
Race and ethnicity						
Asian	0.86 (9.26)	0.89 (9.41)	-0.0031	0.83 (9.10)	0.89 (9.42)	-0.0065
Hispanic	5.54 (22.87)	5.22 (22.25)	0.0141	5.21 (22.22)	5.32 (22.44)	-0.0048
Non-Hispanic Black	8.94 (28.53)	7.86 (26.91)	0.039	8.39 (27.72)	8.13 (27.33)	0.0095
Non-Hispanic White	56.70 (49.55)	53.03 (49.91)	0.0738	56.62 (49.56)	54.60 (49.79)	0.0406
Other[b]	1.04 (10.14)	1.02 (10.05)	0.0017	1.00 (9.95)	1.01 (10.01)	-0.0013
Missing	26.92 (44.35)	53.03 (49.91)	-0.1111	27.95 (44.88)	30.05 (45.85)	0.0406
Annual household income, $						
<30 000	12.32 (32.87)	10.50 (30.65)	0.0573	11.63 (32.05)	10.72 (30.94)	0.0287
30 000-49 999	10.07 (30.09)	8.53 (27.94)	0.053	9.53 (29.36)	8.83 (28.37)	0.0244
50 000-74 999	13.76 (34.45)	11.66 (32.10)	0.063	12.88 (33.50)	12.36 (32.91)	0.0157
75 000-99 999	13.31 (33.97)	12.07 (32.58)	0.0374	13.19 (33.84)	12.47 (33.04)	0.0215
≥ 100 000	24.70 (43.13)	26.35 (44.05)	-0.0378	25.88 (43.80)	26.66 (44.22)	-0.0178
Missing	25.84 (43.77)	30.89 (46.20)	-0.1122	26.89 (44.34)	28.96 (45.36)	-0.0461
Education						
High school or less	19.22 (39.40)	16.15 (36.80)	0.0804	18.22 (38.60)	16.86 (37.44)	0.0358
Some college	33.69 (47.26)	30.73 (46.14)	0.0634	32.91 (46.99)	31.76 (46.56)	0.0246
Associate degree or higher	20.33 (40.24)	21.41 (41.02)	-0.0266	21.05 (40.76)	21.59 (41.14)	-0.0132
Missing	26.77 (44.27)	31.71 (46.54)	-0.1089	27.82 (44.81)	29.79 (45.73)	-0.0435

Note: PSM regression also adjusted for ZIP code dummies. Sample size for patients with chronic or non-chronic conditions reflect the total number of preventive services received. SMD, standardized mean difference.

[a]The analytic sample contains individuals from ages 18-64.

[b]Other race and ethnicity includes any patient not in the listed primary categories.

[c]Zip code 1-99 between cohorts are sufficiently balanced.

© 2026 Tran A et al. *JAMA Network Open*.

eTable 5. Distribution of Per Capita and Per Visit OOP Costs for Preventive Services Among Patients with Chronic Conditions vs. Non-Chronic Conditions (Conditional on Incurring an OOP Cost)

Percentile	Per Capita Level[a]		Per Visit Level[b]	
	Chronic Conditions OOP Cost (USD)	Non-chronic Conditions OOP Cost (USD)	Chronic Conditions OOP Cost (USD)	Non-chronic Conditions OOP Cost (USD)
25th Percentile	$6.66	$7.89	$4.69	$6.13
Median (50th Percentile)	$22.07	$26.40	$15.96	$21.18
75th Percentile	$53.24	$76.87	$35.80	$49.25
90th Percentile	$160.00	$244.29	$103.07	$160.78
99th Percentile	$1,293.76	$1,796.02	$910.96	$1,267.72

Dollar amounts are expressed in 2020 USD.

[a]Preventive services were grouped at the patient level using patient ID.

[b]Preventive services were grouped at the visit level by date of service.

© 2026 Tran A et al. *JAMA Network Open*.

eFigure 1. Magnitude of Unexpected OOP Cost for Preventive Services Between Individuals with Chronic Conditions vs. Non-chronic Conditions

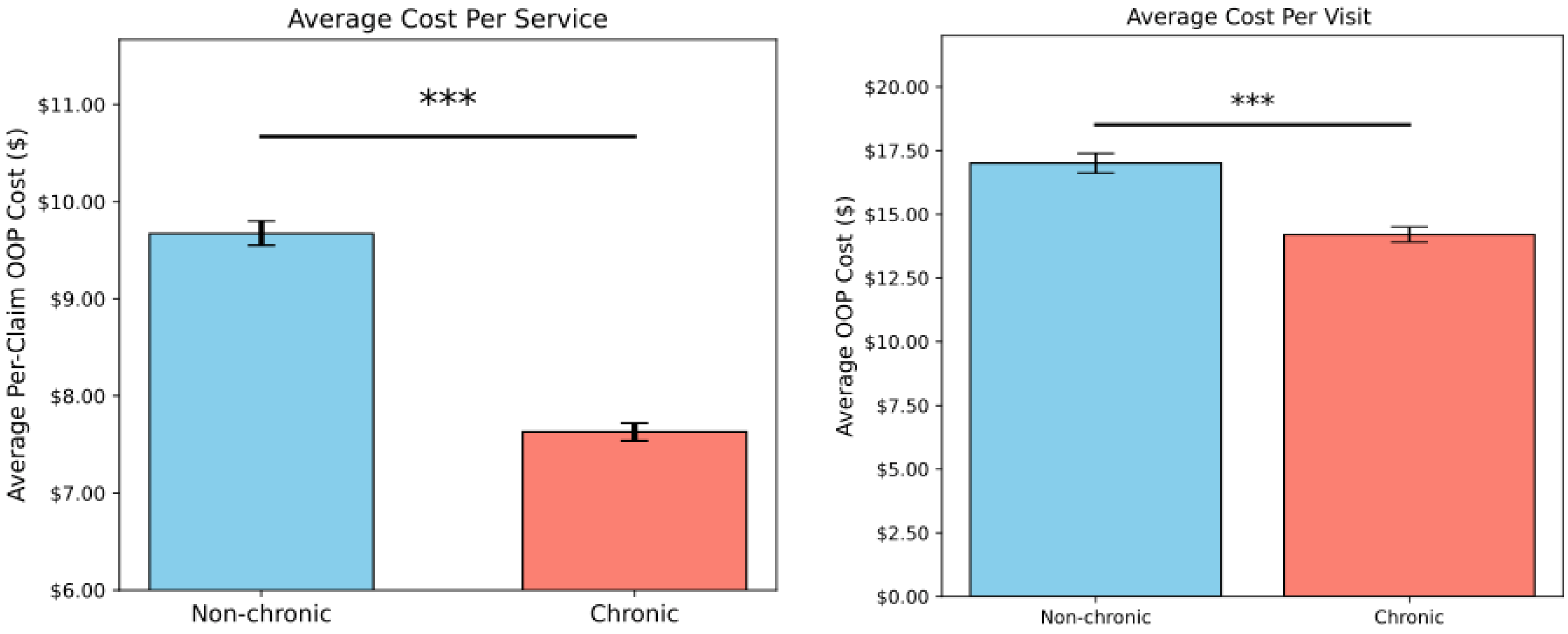

Graphs shows the estimates before PSM-adjustment for unexpected OOP costs for preventive services between individuals with chronic condition versus those without chronic conditions (includes services with no OOP costs). Panel A-B represents the average unexpected OOP costs for preventive care by service and visit, respectively (dollar amounts are expressed in 2020 USD). Error bars denote 95% CIs.

© 2026 Tran A et al. *JAMA Network Open*.

eTable 6. Distribution of Per Capita and Per Visit OOP Unexpected Cost for Preventive Services Among Patients with Chronic Conditions vs. Non-chronic Conditions (Unconditional on Incurring an OOP Cost)

Percentile	Per-Capita Level[a]		Per Visit Level[b]	
	Chronic Conditions OOP Cost (USD)[c]	Non-Chronic Conditions OOP Cost (USD)[c]	Chronic Conditions OOP Cost (USD)[c]	Non-Chronic Conditions OOP Cost (USD)[c]
25th Percentile	$0.00	$0.00	$0.00	$0.00
Median (50th Percentile)	$0.00	$0.00	$0.00	$0.00
75th Percentile	$4.21	$0.00	$0.00	$0.00
90th Percentile	$37.76	$31.55	$20.61	$20.61
99th Percentile	$482.48	$559.46	$244.41	$334.51

[a]Preventive services were grouped at the patient level using patient ID.
[b]Preventive services were grouped at the visit level by date of service.
[c]Dollar amounts are expressed in 2020 USD.

© 2026 Tran A et al. *JAMA Network Open*.

eFigure 2. Aggregate Unexpected OOP Cost Burden by Preventive Service Type Between Individuals with Chronic Conditions vs. Non-chronic Conditions

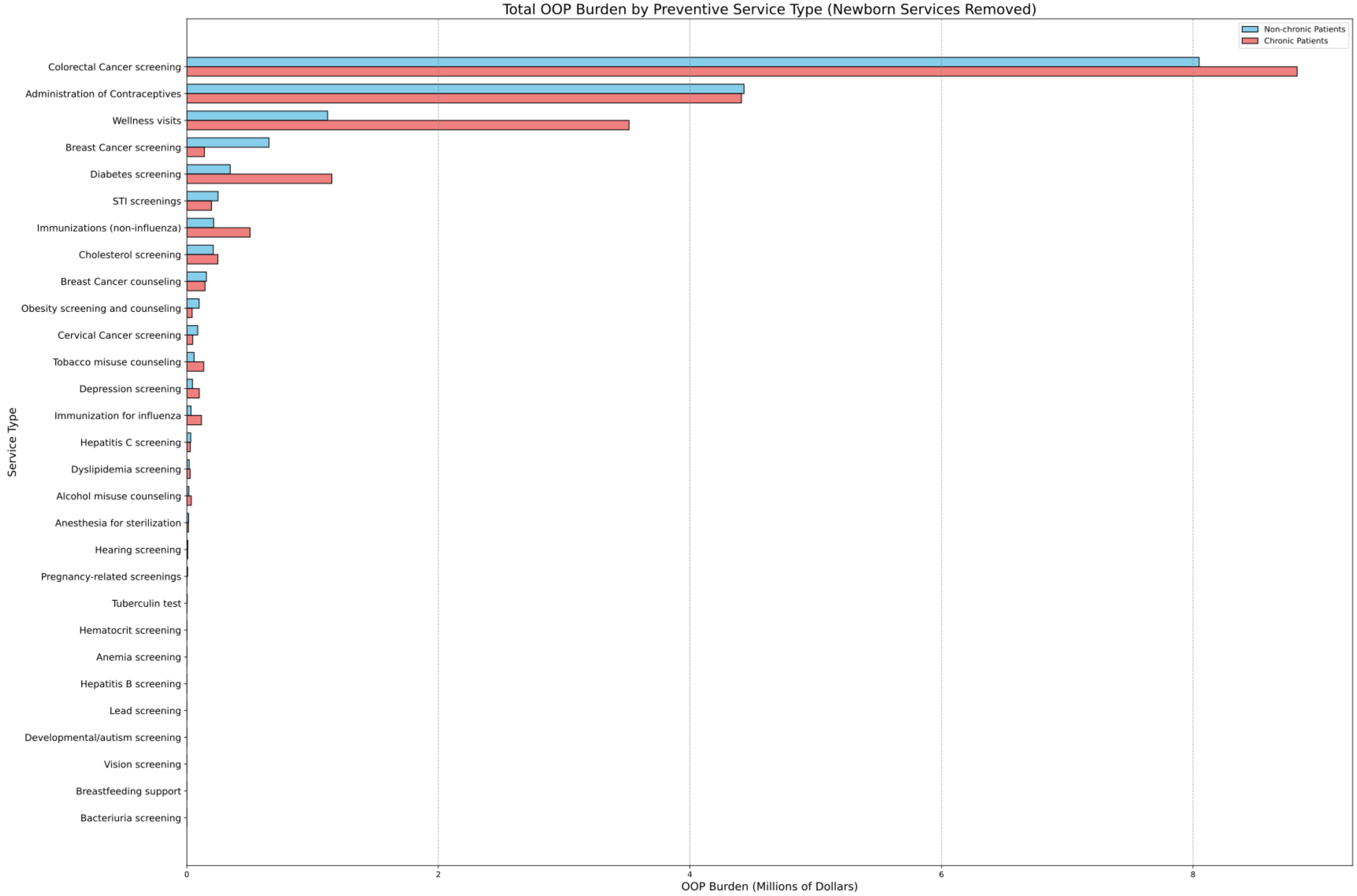

Graphs shows the aggregate estimates before PSM-adjustment for unexpected OOP costs for each preventive service type between individuals with chronic condition versus those without chronic conditions (excludes services with no OOP costs). Unexpected OOP costs are averaged across all individuals for each preventive service type and reported in 2020 USD.

© 2026 Tran A et al. *JAMA Network Open*.

eFigure 3. Per-capita Unexpected OOP Cost Burden by Preventive Service Type Between Individuals with Chronic Conditions vs. Non-chronic Conditions

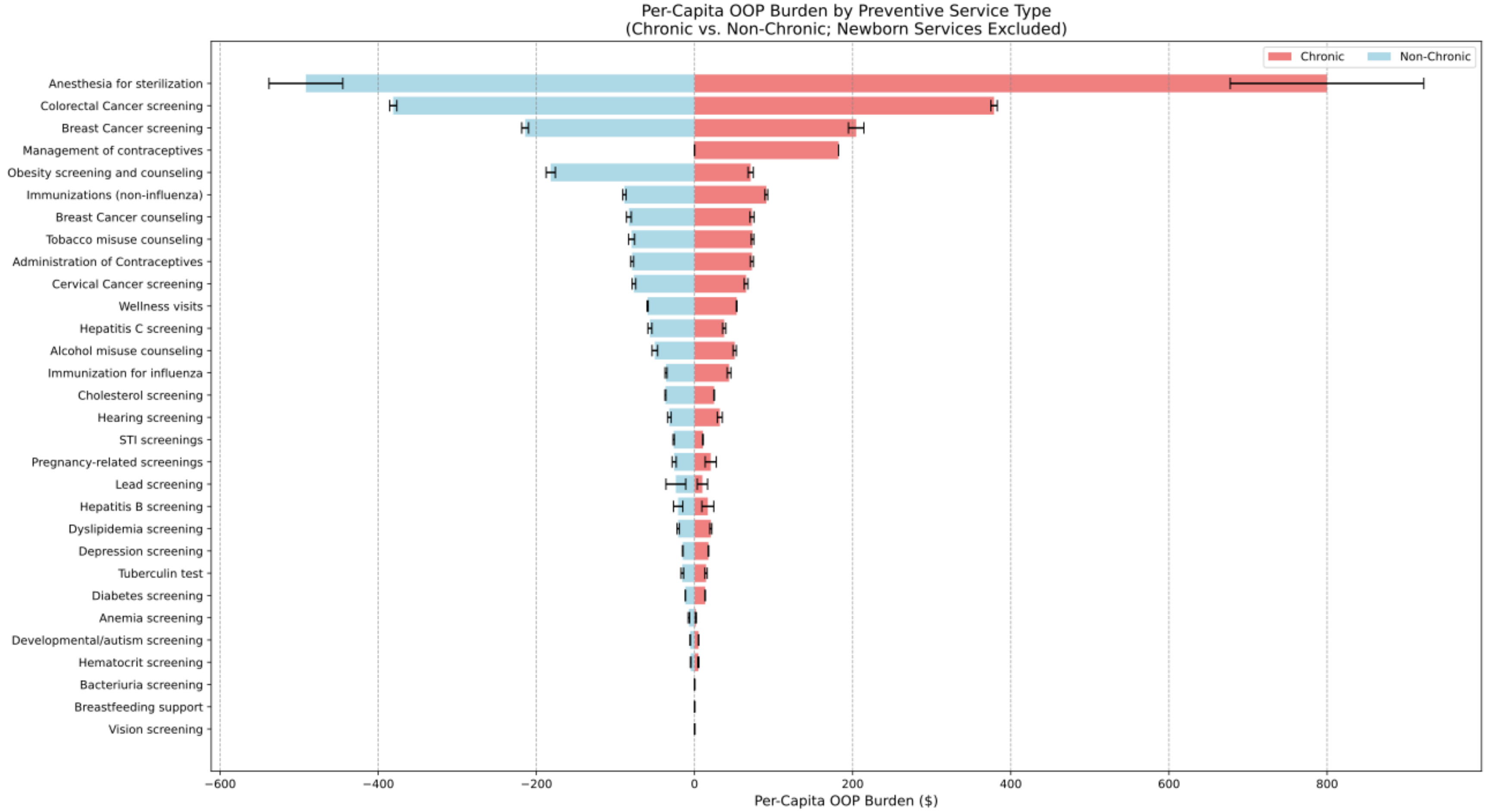

Graphs shows the per-capita estimates before PSM-adjustment for unexpected OOP costs for each preventive service type between individuals with chronic condition versus those without chronic conditions (excludes services with no OOP costs). Unexpected OOP costs are averaged across all individuals for each preventive service type and reported in 2020 USD.

eFigure 4. Differences in Percentage of Preventive Services Incurring an Unexpected OOP Cost for Preventive Care Between Individuals with Chronic Conditions vs. Non-Chronic Conditions

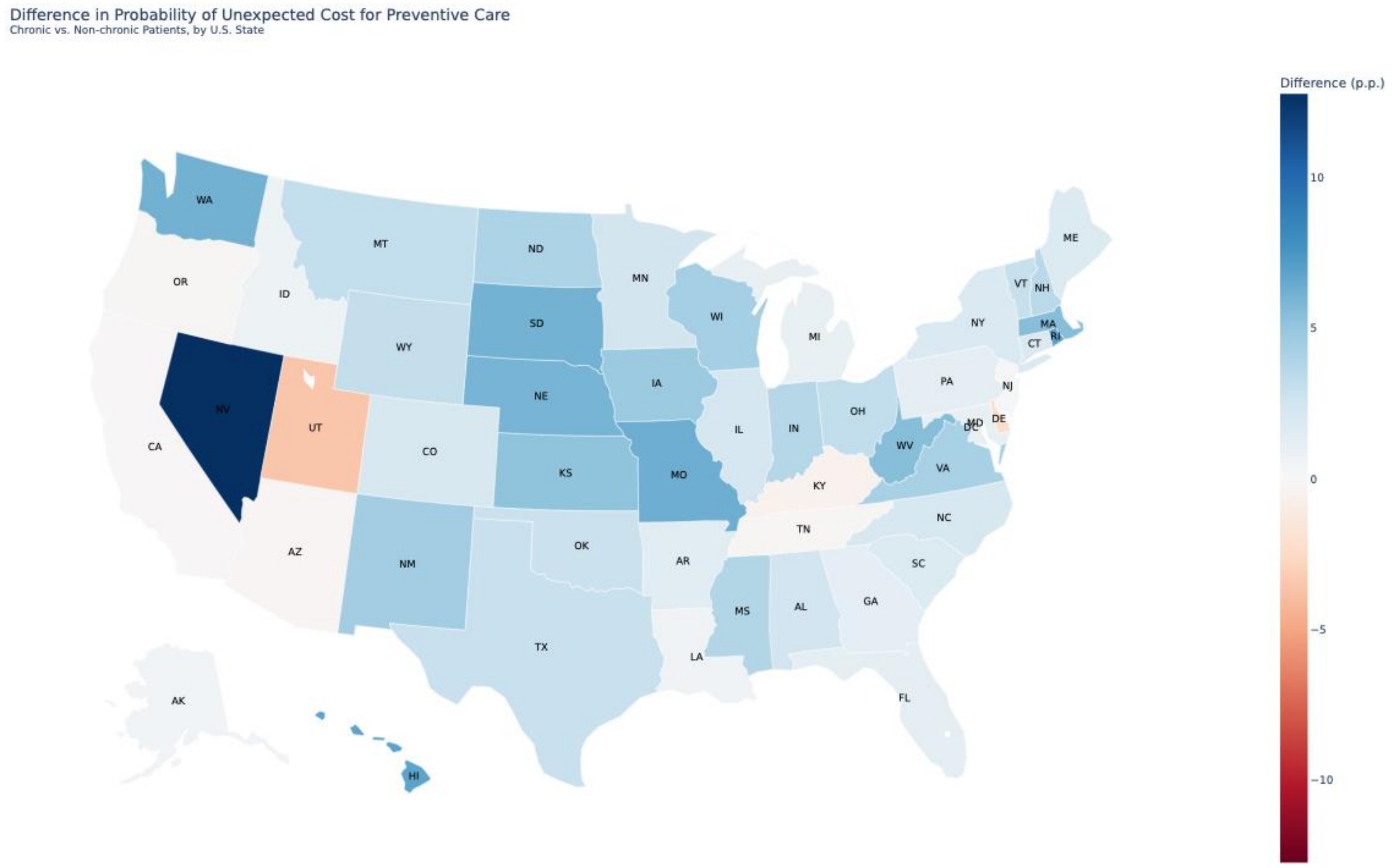

Map displays percentages before PSM-adjustment for unexpected OOP costs for preventive services incurring an unexpected OOP cost for preventive care between individuals with and without chronic conditions across the US. Differences are expressed in percentage points.

© 2026 Tran A et al. *JAMA Network Open*.

eTable 7. Likelihood and Monetary Difference in Unexpected OOP Costs for Preventive Care on Unmatched Sample, Chronic versus Non-chronic (Conditional on Incurring an OOP Cost)

Characteristics	Model (1)		Model (2)	
	Logistic Regression, MEM (95%CI)	p value	Poisson Regression, MEM (95% CI)	p value
Non-chronic vs. Chronic	0.0299 (0.029, 0.031)	<.001	-1.4462 (-1.451 to -1.441)	<.001
Age	-0.0011 (-0.001 to -0.001)	<.001	-0.0632 (-0.063 to -0.0630)	<.001
Sex				
Male	-0.0030 (-0.004 to -0.002)	<.001	1.1459 (1.141, 1.151)	<.001
Female	1 (Reference)		1 (Reference)	
Annual patient income, $				
<30,000	-0.0037 (-0.005 to -0.002)	<.001	-1.5606 (-1.570 to -1.551)	<.001
30,000-49,999	-6.506e-05 (-0.001, 0.001)	.93	-1.1247 (-1.135 to -1.115)	<.001
50,000-74,999	0.0047 (0.003, 0.006)	<.001	-0.6433 (-0.652 to -0.635)	<.001
75,000-99,999	0.0022 (0.001, 0.003)	<.001	-0.6668 (-0.675 to -0.658)	<.001
≥ 100 000	1 (Reference)		1 (Reference)	
Patient Education				
High school or less	0.0128 (0.012, 0.014)	<.001	0.2223 (0.213, 0.231)	<.001
Some college	0.0077 (0.007, 0.009)	<.001	-0.0377 (-0.045 to -0.030)	<.001
Associate's degree or higher	1 (Reference)		1 (Reference)	
Patient race and ethnicity				
Asian	-0.0092 (-0.013 to -0.005)	<.001	-1.3669 (-1.395, -1.339)	<.001
Hispanic	-0.0058 (-0.007 to -0.004)	<.001	-0.7285 (-0.740 to -0.717)	<.001
Non-Hispanic Black	-0.0007 (-0.002, 0.001)	.27	-0.4639 (-0.473 to -0.455)	<.001
Non-Hispanic White	1 (Reference)		1 (Reference)	
Other[b]	0.0081 (0.005, 0.012)	<.001	0.1323 (0.108, 0.156)	<.001

Abbreviations: MEM, marginal effect at the mean; CI, confidence interval

© 2026 Tran A et al. *JAMA Network Open*.

[a]The analytic sample contains individuals from ages 18-64.
[b]Other race and ethnicity includes any patient not in the listed primary categories.

© 2026 Tran A et al. *JAMA Network Open*.

eTable 8. Likelihood and Monetary Difference in OOP Cost for Preventive Care on Matched Sample, Selected Ambulatory Sensitive Conditions (Asthma and Diabetes) vs. Non-chronic Conditions (Conditional on Incurring an OOP Cost)

Chronic Condition	Asthma				Diabetes			
	Logistic Regression, MEM (95%CI)	p value	Poisson Regression, MEM (95%CI)	p value	Logistic Regression, MEM (95%CI)	p value	Poisson Regression, MEM (95%CI)	p value
Selected Ambulatory Care Sensitive Conditions vs. Non-chronic Conditions	0.0668 (0.061, 0.072)	<.001	-2.7158 (-2.747 to -2.685)	<.001	0.071 (0.068, 0.074)	<.001	-4.545 (-4.545 to -4.513)	<.001
Age[a]								
Sex	-0.0355 (-0.043 to -0.028)	<.001	1.3675 (1.328, 1.407)	<.001	0.0016 (-0.001, 0.005)	0.32	1.2848 (1.269, 1.301)	<.001
Male	1 (Reference)		1 (Reference)		1 (Reference)		1 (Reference)	
Female								
Annual patient income, $	-0.0005 (-0.012, 0.011)	.939	-2.0292 (-2.097 to -1.962)	<.001	-0.0092 (-0.015 to -0.003)	.003	-0.8904 (-0.923 to -0.858)	<.001
<30,000	0.0124 (0.000, 0.025)	.046	-1.4446 (-1.515 to -1.374)	<.001	-0.0040 (-0.010, 0.002)	.21	-0.0777 (-0.110 to -0.046)	<.001
30,000-49,999	0.0180 (0.007, 0.029)	.001	-0.5260 (-0.586 to -0.466)	<.001	0.0005 (-0.005, 0.006)	.87	-0.0647 (-0.094 to -0.035)	<.001
50,000-74,999	0.0102 (-0.000, 0.021)	.053	-0.0916 (-0.148 to -0.036)	.001	0.0036 (-0.002, 0.009)	.21	-0.0819 (-0.112 to -0.052)	<.001
75,000-99,999	1 (Reference)		1 (Reference)		1 (Reference)		1 (Reference)	
≥ 100 000								

© 2026 Tran A et al. *JAMA Network Open*.

eTable 8. Likelihood and Monetary Difference in OOP Cost for Preventive Care on Matched Sample, Selected Ambulatory Sensitive Conditions (Asthma and Diabetes) vs. Non-chronic Conditions (Conditional on Incurring an OOP Cost)

Chronic Condition	Asthma				Diabetes			
	Logistic Regression, MEM (95%CI)	p value	Poisson Regression, MEM (95%CI)	p value	Logistic Regression, MEM (95%CI)	p value	Poisson Regression, MEM (95%CI)	p value
Patient Education	0.0036 (-0.008, 0.015)	.53	-0.0567 (-0.121, 0.007)	.08	0.0126 (0.006, 0.019)	<.001	0.4584 (0.426, 0.491)	<.001
High school or less	0.0041 (-0.005, 0.013)	.37	0.1843 (0.135, 0.233)	<.001	0.0075 (0.002, 0.013)	.005	0.6167 (0.589, 0.645)	<.001
Some college	1 (Reference)		1 (Reference)		1 (Reference)		1 (Reference)	
Associate's degree or higher								
Patient race and ethnicity	-0.0171 (-0.054, 0.020)	.36	-0.9804 (-1.200 to -0.761)	<.001	0.0004 (-0.021, 0.022)	.97	-1.9867 (-2.123 to -1.851)	<.001
Asian	0.0096 (-0.005, 0.024)	.19	0.6313 (0.553, 0.709)	<.001	-0.0119 (-0.019 to -0.004)	.002	-0.5927 (-0.633 to -0.552)	<.001
Hispanic	-0.0060 (-0.018, 0.006)	.31	0.1902 (0.124, 0.256)	<.001	0.0035 (-0.002, 0.009)	.21	0.2786 (0.250, 0.307)	<.001
Non-Hispanic Black	1 (Reference)		1 (Reference)		1 (Reference)		1 (Reference)	
Non-Hispanic White	-0.0287 (-0.066, 0.008)	.13	-3.2182 (-3.476 to -2.960)	<.001	-0.0046 (-0.022, 0.013)	.61	-0.8742 (-0.976 to -0.773)	<.001
Other[b]	0.0668 (0.061, 0.072)	<.001	-2.7158 (-2.747 to -2.685)	<.001	0.071 (0.068, 0.074)	<.001	-4.545 (-4.545 to -4.513)	<.001

Abbreviations: MEM, marginal effect at the mean; CI, confidence interval

[a]Coefficient estimates are derived from PSM-adjusted models and reflect marginal effects at the mean.

[b]The analytic sample contains individuals from ages 18-65.

[c]Other race and ethnicity includes any patient not in the listed primary categories.

© 2026 Tran A et al. *JAMA Network Open*.

eTable 9. Likelihood and Monetary Difference in OOP Costs for Preventive Care on Matched Sample, Chronic versus Non-chronic (Years 2017-2019)

Characteristics	Model (1)[a,b] Logistic Regression, MEM (95%CI) N = 3 160 391	p value	Model (2)[a,c] Poisson Regression, MEM (95% CI) N = 3 160 391	p value
Non-chronic vs. Chronic	0.0277 (0.027, 0.028)	<.001	-1.3789 (-1.556 to -1.202)	<.001
Age[d]	-0.0012 (-0.001 to -0.001)	<.001	-0.0592 (-0.067 to -0.052)	<.001
Sex				
Male	-0.0077 (-0.009 to -0.007)	<.001	1.1948 (1.013, 1.377)	<.001
Female	1 (Reference)		1 (Reference)	
Annual patient income, $				
<30,000	0.0008 (-0.001, 0.002)	.33	-1.2391 (-1.602 to -0.876)	<.001
30,000-49,999	0.0055 (0.004, 0.007)	<.001	-1.0892 (-1.374 to -0.805)	<.001
50,000-74,999	0.0083 (0.007, 0.010)	<.001	-0.4304 (-0.842 to -0.019)	.040
75,000-99,999	0.0042 (0.003, 0.006)	<.001	-0.7246 (-0.960 to -0.489)	<.001
≥ 100 000	1 (Reference)		1 (Reference)	
Patient Education				
High school or less	0.0152 (0.014, 0.017)	<.001	0.3283 (-0.116, 0.772)	.15
Some college	0.0082 (0.007, 0.009)	<.001	0.0872 (-0.110, 0.284)	.39
Associate's degree or higher	1 (Reference)		1 (Reference)	
Patient race and ethnicity				
Asian	-0.0069 (-0.011, -0.002)	.003	-0.7127 (-1.285 to -0.140)	.02
Hispanic	-0.0039 (-0.006, -0.002)	<.001	-0.6025 (-1.004 to -0.201)	.003
Non-Hispanic Black	0.0010 (-0.001, 0.003)	.22	-0.3749 (-0.720 -0.029)	.03
Non-Hispanic White	1 (Reference)		1 (Reference)	
Other	0.007 (0.003, 0.011)	.001	0.4117 (-0.172, 0.996)	.17

© 2026 Tran A et al. *JAMA Network Open*.

Abbreviations: MEM, marginal effect at the mean; CI, confidence interval
[a]Coefficient estimates are derived from PSM-adjusted models and reflect marginal effects at the mean.
[b]The outcome in column (1) is an indicator for whether any OOP was incurred at the service level.
[c]The outcome in column (2) is a continuous measure of patient OOP incurred, unconditional on OOP > 0.
[d]The analytic sample contains individuals from ages 18-64.
[e]Other race and ethnicity includes any patient not in the listed primary categories.

© 2026 Tran A et al. *JAMA Network Open*.

eFigure 5. Percentage Distribution of Preventive Service Classification Between Individuals with Chronic Condition vs. Non-chronic Condition

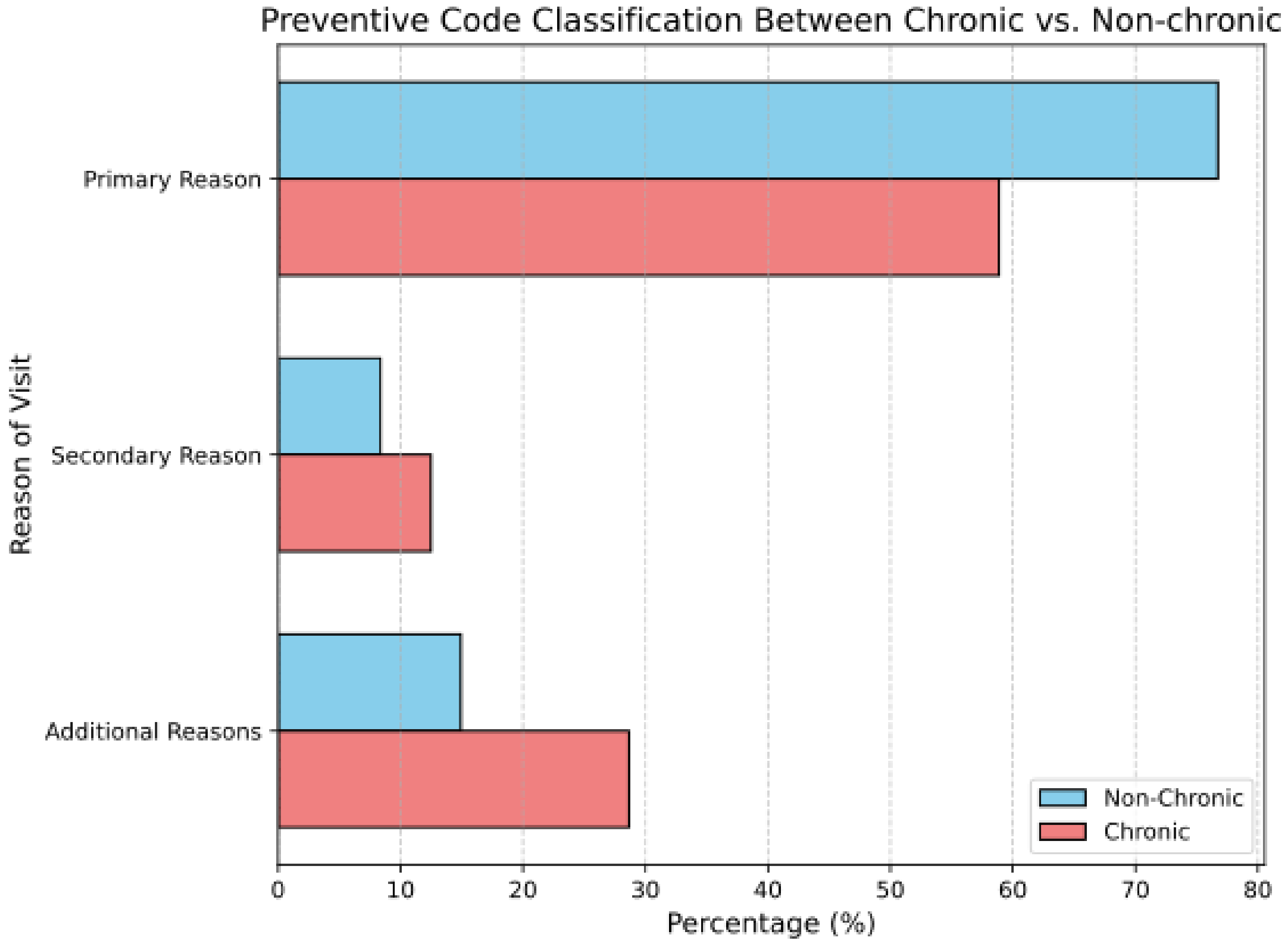

Graphs shows the percentage distribution after PSM-adjustment for preventive service classification between individuals with chronic condition versus those without chronic conditions. Preventive care classification is determined by the positioning of procedure codes within the claim.

© 2026 Tran A et al. *JAMA Network Open*.

eTable 10. Likelihood of Unexpected OOP cost Between High- vs. Low-Complexity Wellness Check on Unmatched Sample (Conditional on Incurring an OOP Cost)

	Logistic Regression[a]			
	High-Complexity Wellness Check, MEM (95% CI)	p value	Incurred OOP cost, MEM (95% CI)	p value
High-Complexity vs. Low-Complexity Wellness Check	-		1.0064 (0.926-1.087)	<.001
Chronic vs. Non-chronic	0.0009 (0.000-0.001)	<.001	-0.0060 (-0.020-0.008)	.40
Age[b]	0.0002*** (0.000-0.000)	<.001	-0.0038 (-0.004 to -0.003)	<.001
Sex				
Male	-0.0004 (-0.001 to -0.000)	.008	0.0027 (-0.012-0.017)	.71
Female	0 (Reference)		0 (Reference)	
Annual patient income, $				
<30,000	0.0004 (-0.000-0.001)	.20	-0.0914 (-0.119 to -0.064)	<.001
30,000-49,999	0.0005 (-0.000-0.001)	.19	-0.0487 (-0.077 to -0.021)	.001
50,000-74,999	0.0005 (-0.000-0.001)	.16	-0.0427 (-0.068 to -0.018)	.001
75,000-99,999	-0.0002 (-0.001-0.000)	.47	-0.0049 (-0.030-0.020)	.71
≥ 100 000	0 (Reference)		0 (Reference)	
Patient Education				
High school or less	5.225e-05 (-0.001-0.001)	.87	-0.0274 (-0.053 to -0.002)	.04
Some college	-3.689e-05 (-0.001-0.000)	.88	-0.0166 (-0.038-0.005)	.13
Associate's degree or higher	0 (Reference)		0 (Reference)	
Patient race and ethnicity				
Asian	0.0007 (-0.001-0.003)	.50	-0.0473 (-0.133-0.038)	.28
Hispanic	0.001 (6.32e-05-0.002)	.04	-0.0617 (-0.092 to -0.032)	<.001
Non-Hispanic Black	-0.0002 (-0.001-0.000)	.63	0.0085 (-0.018-0.035)	.52
Non-Hispanic White	0 (Reference)		0 (Reference)	
Other[c]	-0.0005 (-0.002-0.001)	.40	-0.0564 (-0.121-0.009)	.09

© 2026 Tran A et al. *JAMA Network Open*.

Abbreviations: MEM, marginal effect at the mean; CI, confidence interval
[b]The analytic sample contains individuals from ages 18-65.
[c]Other race and ethnicity includes any patient not in the listed primary categories.

© 2026 Tran A et al. *JAMA Network Open*.
