# Supplementary material for: Role of Chronic Conditions in Out-of-Pocket Costs for Preventive Care in the US
Source: JAMA Netw Open. 2026 Jan 8;9(1):e2553157. doi: 10.1001/jamanetworkopen.2025.53157 (PMC12784232; doi:10.1001/jamanetworkopen.2025.53157)
Supplement: Supplement 3. — Data Sharing Statement [file jamanetwopen-e2553157-s003.pdf]

Data Sharing Statement

Tran. Role of Chronic Conditions in Out-of-Pocket Costs for Preventive Care in the US. *JAMA Netw Open*. Published January 08, 2026. doi:10.1001/jamanetworkopen.2025.53157

Data
Data available: No

Additional Information
Explanation for why data not available: Data is proprietary and we cannot share it publicly per our DUA.
